# MTCH2 Suppresses Thermogenesis by Regulating Autophagy in Adipose Tissue

**DOI:** 10.1002/advs.202416598

**Published:** 2025-03-07

**Authors:** Xin‐Yuan Zhao, Ben‐Chi Zhao, Hui‐Lin Li, Ying Liu, Bei Wang, An‐Qi Li, Tian‐Shu Zeng, Hannah Xiaoyan Hui, Jia Sun, Domagoj Cikes, Nele Gheldof, Jorg Hager, Jian‐Xun Mi, D. Ross Laybutt, Yin‐Yue Deng, Yan‐Chuan Shi, G. Gregory Neely, Qiao‐Ping Wang

**Affiliations:** ^1^ Laboratory of Metabolism and Aging School of Pharmaceutical Sciences (Shenzhen) Shenzhen Campus of Sun Yat‐sen University Shenzhen 518107 China; ^2^ Wuhan Union Hospital Huazhong University of Science and Technology Wuhan 430022 China; ^3^ School of Biomedical Sciences The Chinese University of Hong Kong Hong Kong 999077 China; ^4^ Department of Endocrinology Zhujiang Hospital Southern Medical University Guangzhou 510280 China; ^5^ Institute of Physiology and Pathophysiology Johannes Kepler University Linz Linz 4020 Austria; ^6^ Ecole Polytechnique de Lausanne (EPFL) Lausanne CH‐1015 Switzerland; ^7^ Nestlé Institute of Health Sciences Lausanne CH‐1015 Switzerland; ^8^ Key Laboratory of Big Data Intelligent Computing Chongqing University of Posts and Telecommunications Chongqing 400065 China; ^9^ Chongqing Key Laboratory of Image Cognition Chongqing University of Posts and Telecommunications Chongqing 400065 China; ^10^ College of Computer Science and Technology Chongqing University of Posts and Telecommunications Chongqing 400065 China; ^11^ Garvan Institute of Medical Research St Vincent's Clinical School UNSW Sydney Darlinghurst Sydney NSW 2010 Australia; ^12^ School of Pharmaceutical Sciences (Shenzhen) Sun Yat‐sen University Shenzhen 518107 China; ^13^ Neuroendocrinology Group Garvan Institute of Medical Research Darlinghurst Sydney NSW 2010 Australia; ^14^ St Vincent's Clinical School Faculty of Medicine University of New South Wales Sydney NSW 2010 Australia; ^15^ The Dr. John and Anne Chong Laboratory for Functional Genomics Charles Perkins Centre and School of Life & Environmental Sciences The University of Sydney Sydney NSW 2006 Australia; ^16^ Guangdong Provincial Key Laboratory of Diabetology Guangzhou Key Laboratory of Mechanistic and Translational Obesity Research The Third Affiliated Hospital of Sun Yat‐sen University Guangzhou 510630 China; ^17^ State Key Laboratory of Anti‐Infective Drug Discovery and Development School of Pharmaceutical Sciences Sun Yat‐sen University Guangzhou 510006 China

**Keywords:** **a**dipose tissue, autophagy, mitochondrial carrier homolog 2 (MTCH2), obesity, thermogenesis

## Abstract

Stimulating adipose tissue thermogenesis has emerged as a promising strategy for combating obesity, with uncoupling protein 1 (UCP1) playing a central role in this process. However, the mechanisms that suppress adipose thermogenesis and energy dissipation in obesity are not fully understood. This study identifies mitochondrial carrier homolog 2 (MTCH2), an obesity susceptibility gene, as a negative regulator of energy homeostasis across flies, rodents, and humans. Notably, adipose‐specific MTCH2 depletion in mice protects against high‐fat‐diet (HFD)‐induced obesity and metabolic disorders. Mechanistically, MTCH2 deficiency promotes energy expenditure by stimulating thermogenesis in brown adipose tissue (BAT) and browning of subcutaneous white adipose tissue (scWAT), accompanied by upregulated UCP1 protein expression, enhanced mitochondrial biogenesis, and increased lipolysis in BAT and scWAT. Using integrated RNA sequencing and proteomic analyses, this study demonstrates that MTCH2 is a key suppressor of thermogenesis by negatively regulating autophagy via Bcl‐2‐dependent mechanism. These findings highlight MTCH2's critical role in energy homeostasis and reveal a previously unrecognized link between MTCH2, thermogenesis, and autophagy in adipose tissue biology, positioning MTCH2 as a promising therapeutic target for obesity and related metabolic disorders. This study provides new opportunities to develop treatments that enhance energy expenditure.

## Graphical abstract

This study demonstrates that MTCH2 acts as an evolutionarily conserved regulator of energy homeostasis across *Drosophila*, rodents, and humans. Adipose‐specific MTCH2 ablation enhances brown/beige adipose thermogenesis through Bcl‐2‐dependent autophagy, promoting energy expenditure and counteracting obesity‐associated metabolic disorders. These findings establish MTCH2 as a pivotal metabolic gatekeeper and propose its therapeutic targeting for obesity treatment via adipose tissue remodeling.

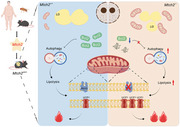

## 1 Introduction

Obesity, characterized by chronic caloric imbalance where intake surpasses expenditure,^[^ ^1^ ^]^ affects approximately one billion individuals worldwide,^[^ ^2^ ^]^ presenting significant challenges for effective weight management. Adipose tissue, traditionally regarded as a passive energy reservoir, is now recognized as a dynamic organ that plays a crucial role in regulating energy homeostasis.^[^ ^3^, ^4^ ^]^ A key process in this regulation isUCP1‐mediated thermogenesis, which dissipates the mitochondrial proton gradient to generate heat and increase energy expenditure.^[^ ^3^, ^5^, ^6^ ^]^ Several key regulators of UCP1 expression and activity have been identified,^[^ ^3^, ^5^, ^7^, ^8^ ^]^ including PR domain zinc finger protein 16 (PRDM16), peroxisome proliferator‐activated receptor γ (PPARγ), PPAR co‐activator 1α (PGC‐1α), fibroblast growth factor 21 (FGF21), and β‐adrenergic signaling pathways. However, the complete molecular mechanisms governing thermogenesis in adipose tissue remain incompletely understood.

While genome‐wide association studies (GWAS) have identified numerous genetic loci associated with obesity,^[^ ^9^,^10^ ^]^ relatively few studies have focused specifically on the regulation of energy balance within adipose tissue.^[^ ^11^, ^12^, ^13^, ^14^ ^]^ This gap underscores the need for targeted investigations to uncover novel adipose‐specific regulators of energy homeostasis. In the DiOGenes study, we previously identified 29 genes in abdominal subcutaneous adipose tissue associated with weight loss during calorie restriction.^[^ ^15^ ^]^ However, the functional roles of these genes in energy regulation remain largely unexplored.

In this study, we employed *Drosophila* as a model organism to investigate the metabolic roles of these candidate genes, identifying several candidate regulators of energy balance. Among them, MTCH2 emerged as a key regulator due to its established links to obesity and energy homeostasis. Through in vivo and in vitro models, coupled with comprehensive metabolic phenotyping and integrative proteomic and transcriptomic analyses, we demonstrate that MTCH2 acts as a suppressor of thermogenesis by negatively regulating autophagy via the Bcl‐2 pathway. Our findings establish MTCH2 as a critical regulator of thermogenesis, offering new insights into its role in energy balance and its therapeutic potential in combating obesity and related metabolic disorders.

## 2 Results

### 2.1 Identification of *MTCH2* as a Conserved Negative Regulator of Energy Homeostasis

**Figure 1:**
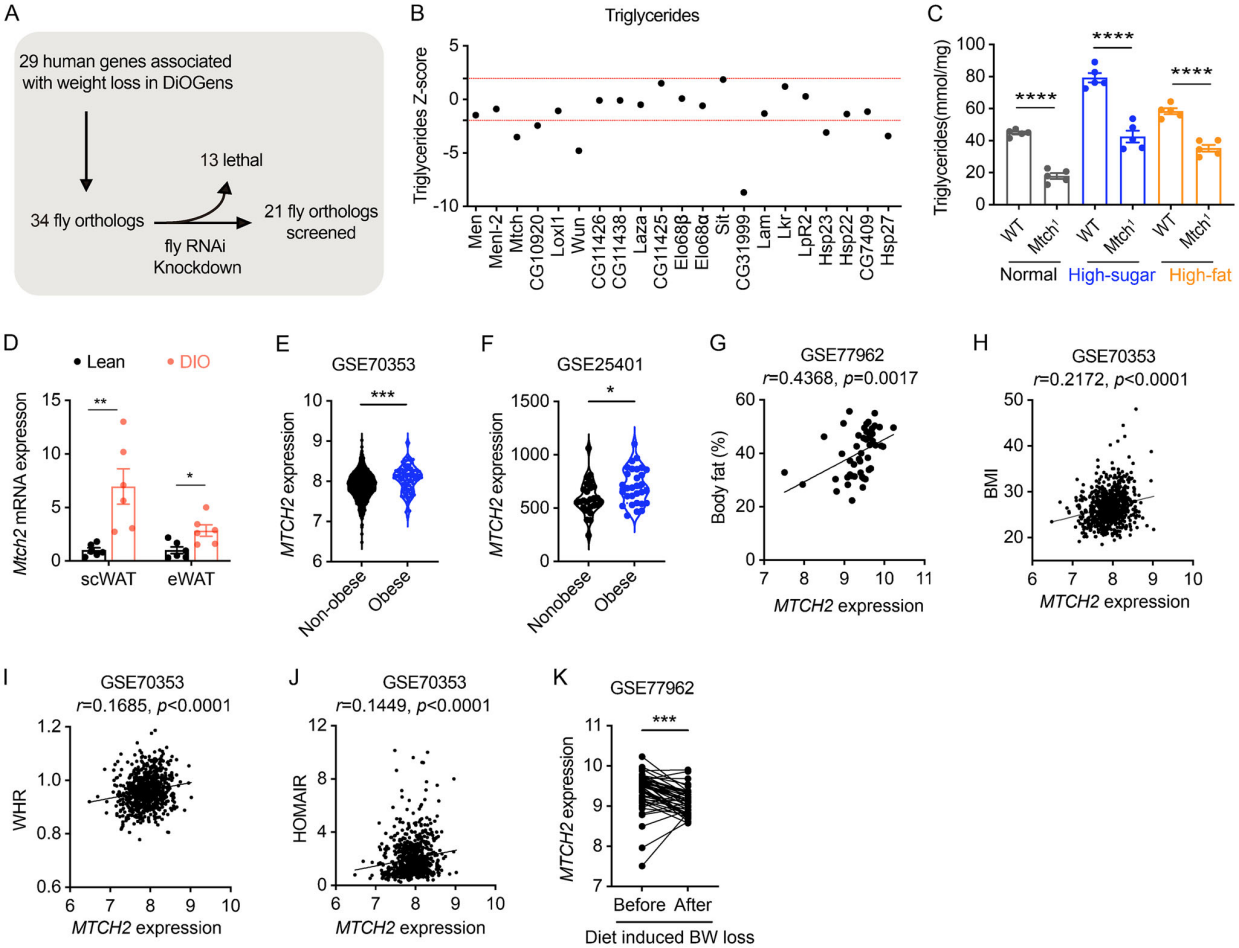
Identification of MTCH2 as a conserved regulator of energy homeostasis. (A) A scheme of functional RNAi knockdown screen of 29 genes associated with weight loss using *Drosophila melanogaster*. (B) Z‐scores of TAG levels for each UAS‐RNAi line were presented. Z≥ 1.96 or ≤−1.96 (red dotted lines indicate threshold) was regarded as significant hits. (C) TAG levels were decreased in *Mtch* mutant flies fed normal, high sucrose or high‐fat diet (*n* = 5 biological sample, 6 flies per sample). (D) *Mtch2* mRNA expression was elevated in mice fed a high‐fat diet (DIO mice) compared to those mice fed a chow (lean mice) (*n* = 6). (E,F) *MTCH2* mRNA expression was lower in the abdominal scWAT of individuals with obesity than those without obesity (*n* = 770 biological samples for E, *n* = 56 for (F). (G–J) A positive correlation existed between *MTCH2* mRNA expression and body fat (G), BMI (H), waist‐to‐hip ratio (WHR) (I), and HOMA‐IR (J) in abdominal scWAT of individuals (*n* = 49 biological samples for G, *n* = 770 biological samples for (H–J). (K) *MTCH2* mRNA expression was significantly reduced in the abdominal scWAT of patients with obese after dietary intervention (*n* = 46 biological samples). Data are represented as mean ± SEM. Two‐tailed unpaired Student's *t*‐test (C–F), The Spearman's rank‐order correlation coefficient (r) (G–J), and two‐tailed paired *t*‐test (K) were used. *p < 0.05, **p < 0.01, ***p < 0.005, ****p < 0.001.

We performed an RNAi screen targeting the 34 *Drosophila* homologs of 29 human genes that were revealed in our previous study to have differential expression in adipose tissue during calorie restriction.^[^ ^15^ ^]^ This screen assessed their impact on fat metabolism in adult flies using the *Actin‐Gal4* driver (**Figure** **1**A). We identified six genes that play a critical role in fat metabolism, with their silenced homologs (6/34, 17.6%) leading to a reduction in TAG levels (Figure 1B). Among these, MTCH2 is of particular interest due to its established role in the regulation of energy homeostasis.^[^ ^16^ ^]^ To validate the RNAi findings, we analyzed *Drosophila Mtch* hypomorphic mutants with a 70% reduction in MTCH2 protein levels (Figure S1A, Supporting Information). TAG levels were markedly reduced in these mutants (Figure 1C). Importantly, TAG levels were significantly lower in *Mtch* mutant flies fed a high‐sugar or high‐fat diet (Figure 1C), demonstrating resistance to obesogenic diets.

To further investigate the role of *MTCH2* in adiposity and energy homeostasis, we examined *Mtch2* expression in adipose tissue and other metabolically important tissues in chow and high‐fat diet (HFD, 60% fat) ‐fed mice. In mice fed a chow diet, *Mtch2* expression was detected in all tissues examined including BAT, scWAT, epididymal WAT (eWAT), retroperitoneal WAT (rWAT), muscle, and liver, with the highest levels found in BAT (Figure S1B, Supporting Information). In HFD‐fed mice, *Mtch2* mRNA expression was significantly upregulated in both scWAT and eWAT compared with chow‐fed mice (Figure 1D), suggesting a positive relationship between *Mtch2* expression and diet‐induced obesity.

To evaluate whether *MTCH2* expression is associated with adiposity and metabolic health in humans, we analyzed publicly available datasets (GSE77962, GSE70353, GSE77962) from the Gene Expression Omnibus (GEO). Consistent with our results in DIO mice, *MTCH2* expression was significantly elevated in scWAT from obese compared with non‐obese individuals (Figure 1E,F). Moreover, *MTCH2* expression in human scWAT was positively correlated with body fat (Figure 1G), body mass index (BMI) (Figure 1H), waist‐to‐hip ratio (WHR) (Figure 1I), and insulin resistance (Figure 1J). Notably, *MTCH2* expression was downregulated following diet‐induced weight loss in patients with obesity (Figure 1K), implying that Mtch2 levels are dependent on the prevailing adiposity and metabolic state. Collectively, these findings reveal *MTCH2* as a conserved obesity‐linked factor in adipose tissue across species, and that elevated *MTCH2* expression in adipose tissue may play a role in the negative regulation of energy homeostasis and metabolic dysfunction such as insulin resistance.

### 2.2 Adipose Tissue‐Specific *Mtch2* Knockout Prevents HFD‐Induced Obesity and Metabolic Disorder

To examine the role of MTCH2 specifically in adipose tissue, we generated adipocyte‐specific *Mtch2* knockout mice (*Mtch2^flox/flox^,Adipoq‐Cre* referred to as *Mtch2^AKO^* hereafter) by crossing *Mtch2^flox/flox^* (*Mtch2^f/f^*) mice with *adiponectin‐Cre* mice (Figure S2A, Supporting Information). The successful deletion of *mtch2* in adipose tissue of *Mtch2^AKO^* mice was confirmed by the presence of a single DNA band at 221 and 204 bp (Figure S2B, Supporting Information). Furthermore, *Mtch2^AKO^* mice exhibited a significant reduction in *Mtch2* mRNA and protein levels in various adipose tissue depots, while its levels in non‐adipose tissues, the liver and skeletal muscle, remained unchanged (Figure S2C,D, Supporting Information), thus confirming the specificity of *Mtch2* deletion in adipose tissue.

**Figure 2:**
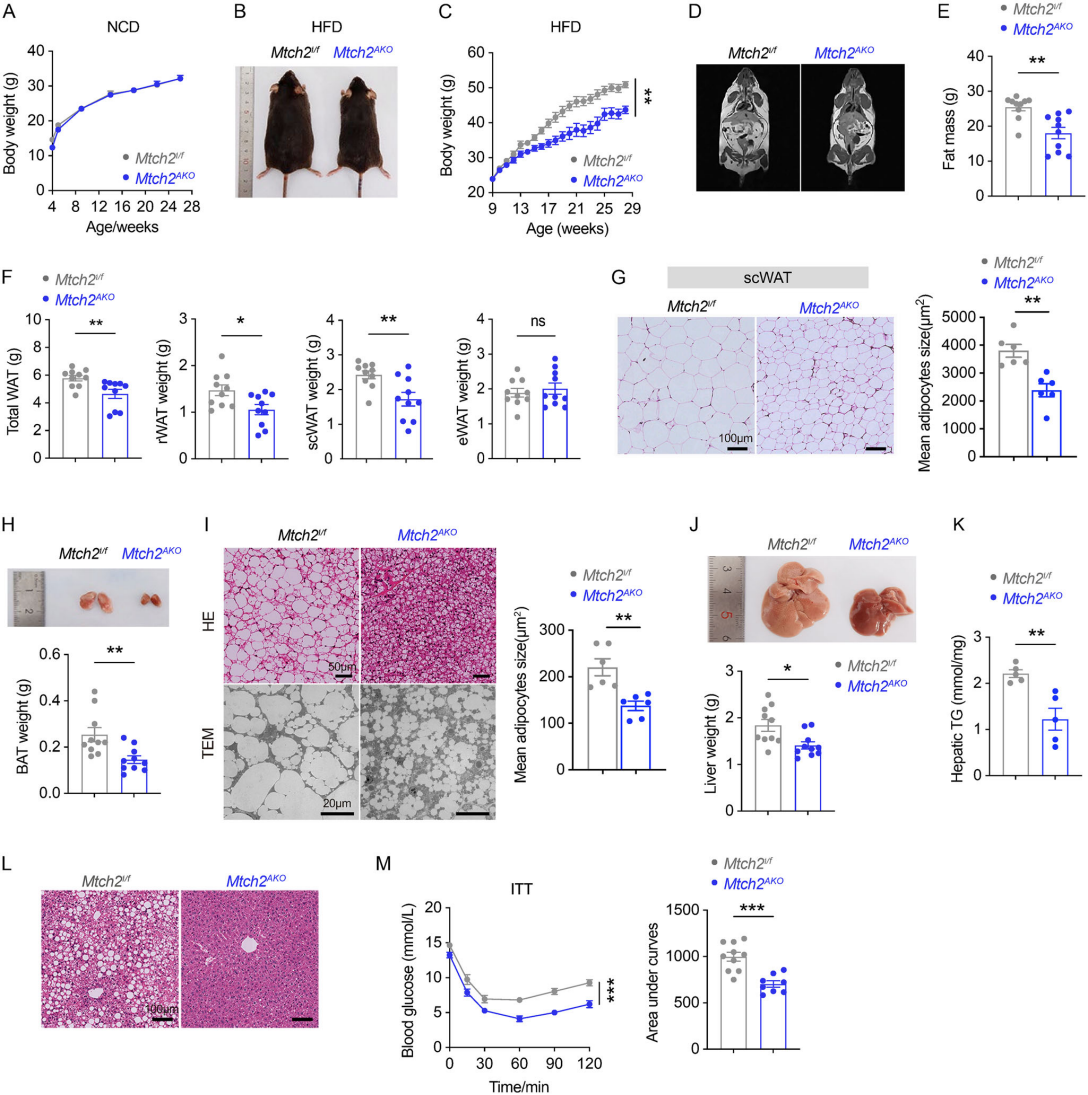
*Mtch2^AKO^* reduces fat depots and hepatic accumulation under HFD. (A) *Mtch2^AKO^* did not affect body weight in mice fed chow (*n* = 8–9). (B,C) Body weight was gained less in *Mtch2^AKO^* mice under HFD feeding for 19 weeks. Representative image (B) and body weight growth curve (*n* = 10, C). (D,E) *Mtch2^AKO^* mice displayed a lower fat composition. Representative NMR analysis image (D) and fat mass composition by NMR (E). (F,G) *Mtch2^AKO^* mice exhibited fewer fat depots. The dissected weight of total WAT, rWAT, scWAT and eWAT (F, *n* = 10), H&E staining and adipocyte size analysis of scWAT (G). (H,I) BAT was reduced in *Mtch2^AKO^* mice. Representative image of dissected BAT (H, *n* = 10), the analyses of H&E staining and TEM on BAT and adipocyte size of BAT in H&E staining (I). (J–L) Fat accumulation was decreased in *Mtch2^AKO^* mice. Representative image of the dissected liver (J, *n* = 10), hepatic TAG levels (K, *n* = 5), and liver H&E staining (L). (M) *Mtch2^AKO^* mice exhibited enhanced insulin sensitivity in mice fed HFD for 18 weeks. Insulin tolerance test and quantified in the area under curves (*n* = 8–10). Data are represented as mean ± SEM. Two‐way ANOVA followed by Bonferroni's multiple comparisons test (C,M), Two‐tailed unpaired *t*‐test was used (E–K,M), *p < 0.05, **p < 0.01, and ***p < 0.001, ns, not significant.

To assess *Mtch2*’s role in energy balance and glucose homeostasis, *Mtch2^AKO^* mice and littermate *Mtch2^f/f^* control mice were fed a chow or HFD for up to 26 weeks. Body weight (BW) was not different between *Mtch2^AKO^* and control mice fed a chow diet (**Figure** **2**A; Figure S3A,B, Supporting Information), suggesting that adipocyte‐specific deletion of *Mtch2* has minimal effects on energy balance under normal dietary conditions. However, when subjected to high‐fat feeding, *Mtch2^AKO^* mice displayed resistance to BW gain, showing a 14.2% reduction compared to controls (Figure 2B,C; Figure S3C,D, Supporting Information). Analysis by magnetic resonance imaging (MRI) revealed that the lower BW gain was accompanied by a 29.2% reduction in fat mass and fat mass percentage in *Mtch2^AKO^* compared to control mice (Figure 2D,E; Figure S3E, Supporting Information). Similarly, dissected weights of total fat depots, scWAT, and rWAT, were significantly reduced in *Mtch2^AKO^* compared to control mice (Figure 2F). Strikingly, H&E staining revealed a higher proportion of relatively small adipocytes in scWAT of *Mtch2^AKO^* compared to control mice (Figure 2G). Similarly, after HFD feeding, BAT weight was reduced by 42.5% in *Mtch2^AKO^* compared to control mice (Figure 2H). Furthermore, H&E staining and transmission electron microscopy (TEM) revealed the presence of smaller adipocytes in BAT from *Mtch2^AKO^* compared to control mice (Figure 2I). These findings indicate that adipocyte expression of *Mtch2* is critical for the long‐term remodeling of adipose tissues in HFD mice.

We next examined the livers of *Mtch2^AKO^* and *Mtch2^f/f^* mice following HFD feeding. The liver weight was reduced by 21.3% and appeared less pale in *Mtch2^AKO^* mice compared to control mice (Figure 2J). This was accompanied by a 35.8% decrease in hepatic TAG content (Figure 2K) and the presence of fewer lipid droplets in the liver (Figure 2L). Moreover, while aspartate aminotransferase (AST) remained unchanged, plasma alanine transaminase (ALT) and the ratio of ALT to AST were significantly reduced in *Mtch2^AKO^* mice (Figure S3F–H, Supporting Information). This systemic reduction in adiposity was associated with improved insulin sensitivity in *Mtch2^AKO^* mice compared to controls (Figure 2M). The area under the curve (AUC) for blood glucose levels from 0 to 120 min during the insulin tolerance test was significantly reduced in *Mtch2^AKO^* mice compared to control mice (Figure 2M). Together, these findings suggest that fat tissue‐specific *Mtch2* deletion not only protects against HFD‐induced fat accumulation in adipose tissue but also reduces liver lipid deposition and enhances whole‐body insulin sensitivity in mice.

### 2.3 Adipose Tissue‐Specific *Mtch2* Knockout Increases Energy Expenditure by Promoting BAT Thermogenesis and scWAT Browning

**Figure 3:**
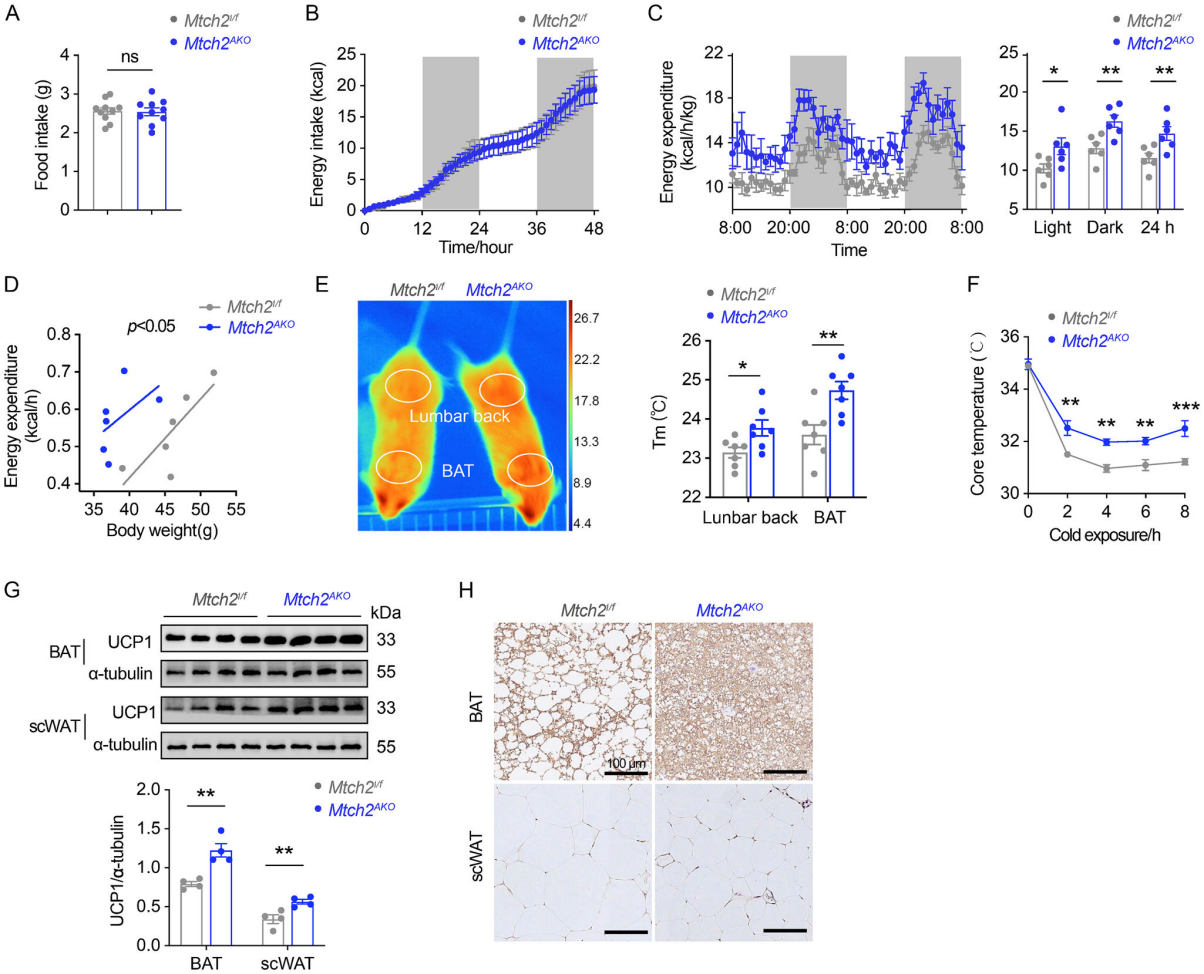
*Mtch2^AKO^* increases energy expenditure by promoting BAT thermogenesis and scWAT browning in HFD‐fed mice. (A,B) Caloric intake was not altered in *Mtch2^AKO^* mice. Daily food intake (A, *n* = 10) and accumulative food intake (B, *n* = 6). (C,D) Energy expenditure was enhanced in *Mtch2^AKO^* mice (*n* = 6). (E,F) *Mtch2^AKO^* mice displayed higher surface temperature and core temperature under cold exposure (4 °C) at the age of 8 weeks. Representative infra‐red thermal image of lumbar back and BAT and qualification of temperatures of them (*n* = 7 for E and 4 for F). (G,H) UCP1 protein levels were elevated in BAT and scWAT of *Mtch2^AKO^* mice as shown in western blot (G, *n* = 4) and IHC staining of UCP1 (H). Data are represented as mean ± SEM. Two‐tailed unpaired *t*‐test (A,C,E–G) or ANCOVA using body weight as covariate (D) was used. *p < 0.05, **p < 0.01, and ***p < 0.001, ns, not significant.

To investigate whether the leaner phenotype observed in *Mtch2^AKO^* mice under HFD feeding results from changes in energy balance, we measured food intake and energy expenditure using metabolic chambers. *Mtch2^AKO^* mice exhibited no significant changes in average daily food intake (**Figure** **3**A) or cumulative energy intake over a 2‐day period (Figure 3B) compared to their littermate controls, indicating that the reduction in BW and fat mass observed in *Mtch2^AKO^* mice was not due to altered calorie intake. In contrast, *Mtch2^AKO^* mice exhibited increased oxygen (O_2_) consumption (Figure S4A, Supporting Information) and carbon dioxide (CO_2_) production (Figure S4B, Supporting Information), resulting in a significant increase in overall energy expenditure (Figure 3C,D). These findings suggest that augmented energy expenditure is likely responsible for the reduced BW gain and fat accretion in *Mtch2^AKO^* mice. Additionally, there were no significant changes in physical activity between the groups (Figure S4C, Supporting Information), excluding altered physical movement as a potential cause for the increased energy expenditure.

A key component of energy expenditure involves body temperature control, with BAT activation important for maintaining core temperature during cold exposure. To explore this, we employed a non‐invasive infra‐red camera to measure skin surface temperature^[^ ^17^ ^]^ under cold conditions. We calculated temperatures above the BAT as well as over the lumbar spine region (Figure 3E). *Mtch2^AKO^* mice displayed significantly higher temperatures in both the lumbar spine region and BAT under cold exposure compared to control mice (Figure 3E). Increased body temperatures were also observed in *Mtch2^AKO^* mice using the conventional method of rectal probes to measure core body temperature over an 8‐h monitoring period (Figure 3F).

Given the critical role of UCP1 in BAT thermogenesis and scWAT browning,^[^ ^5^, ^18^ ^]^ we examined the UCP1 protein levels in BAT and scWAT of HFD‐fed mice. UCP1 levels were significantly upregulated in both BAT and scWAT of *Mtch2^AKO^* mice compared to control mice (Figure 3G). Immunohistochemical staining further revealed abundant UCP1 expression in smaller, multilocular adipocytes within the BAT of *Mtch2^AKO^* mice (Figure 3H). Additionally, clusters of UCP1‐positive adipocytes were detected in the scWAT of *Mtch2^AKO^* mice (Figure 3H), indicating that *Mtch2* deletion promotes the development of beige fat. Collectively, these results suggest that adipose tissue‐specific deletion of *Mtch2* enhances energy expenditure under HFD condition through the promotion of BAT thermogenesis and the conversion of scWAT into energy‐burning beige WAT via upregulation of UCP1.

### 2.4 Adipose Tissue‐Specific *Mtch2* Knockout Increases Mitochondrial Biogenesis and Oxidative Phosphorylation in Adipose Tissues

**Figure 4:**
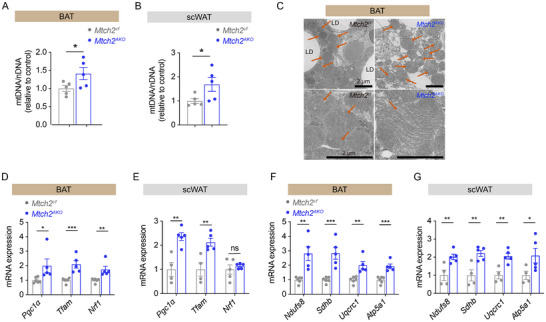
*Mtch2^AKO^* increases mitochondrial biogenesis and oxidative phosphorylation in adipose tissues of HFD‐fed mice. (A,B) The copies of mtDNA were increased in BAT (A) and scWAT (B) of *Mtch2^AKO^* mice by qPCR qualification (*n* = 5). (C) The number and structure of mitochondria were increased in BAT of *Mtch2^AKO^* by TEM analysis. (D,E) Mitochondrial biogenesis was boosted in BAT (D, *n* = 5) and scWAT (E, *n* = 4–5) of *Mtch2^AKO^* mice by qPCR analysis of typic markers. (F,G) Oxidative phosphorylation was enhanced in BAT (F, *n* = 5) and scWAT (G, *n* = 4–5) of *Mtch2^AKO^* mice by qPCR analysis of typic markers. Data are represented as mean ± SEM. Two‐tailed unpaired *t*‐test was used (A,B,D–G). *p < 0.05, **p < 0.01, and ***p < 0.001, ns, not significant.

We next assessed potential mechanisms for the changes in fat thermogenesis in *Mtch2^AKO^* mice by examining mitochondrial features. *Mtch2* knockout led to a significant increase in mitochondrial DNA (mtDNA) copy number in BAT (**Figure** **4**A) and scWAT (Figure 4B), indicating an increase in mitochondrial abundance. Furthermore, TEM analysis revealed that BAT adipocytes from *Mtch2^AKO^* mice displayed an increased number of mitochondria with more pronounced cristae structures compared to control mice (Figure 4C). These findings uncover a novel regulatory function for *Mtch2* in mitochondrial biogenesis in BAT and scWAT.

We next examined changes in the expression of genes involved in mitochondrial biogenesis. The expression of *Pgc1α*, *Tfam*, and *Nrf1* were significantly increased in both BAT (Figure 4D) and scWAT (Figure 4E) of *Mtch2^AKO^* mice compared to control mice. We next assessed changes in the expression of genes critical for mitochondrial oxidative phosphorylation. The expression of *Ndufs8*, *Sdhb*, *Uqcrc1*, and *Atp5a1* were markedly upregulated in BAT (Figure 4F) and scWAT (Figure 4G) of *Mtch2^AKO^* mice compared to control mice. These findings suggest *Mtch2* deficiency promotes mitochondrial biogenesis and augments mitochondrial function in adipose tissues. Taken together, the results suggest that the enhanced thermogenesis and energy expenditure observed following *Mtch2* deletion is driven by increases in mitochondrial abundance, function and uncoupling of the electron transport chain.

### 2.5 Proteomics and Transcriptomics Analyses Reveal that *Mtch2* Deficiency Enhances Thermogenesis and Autophagy

**Figure 5:**
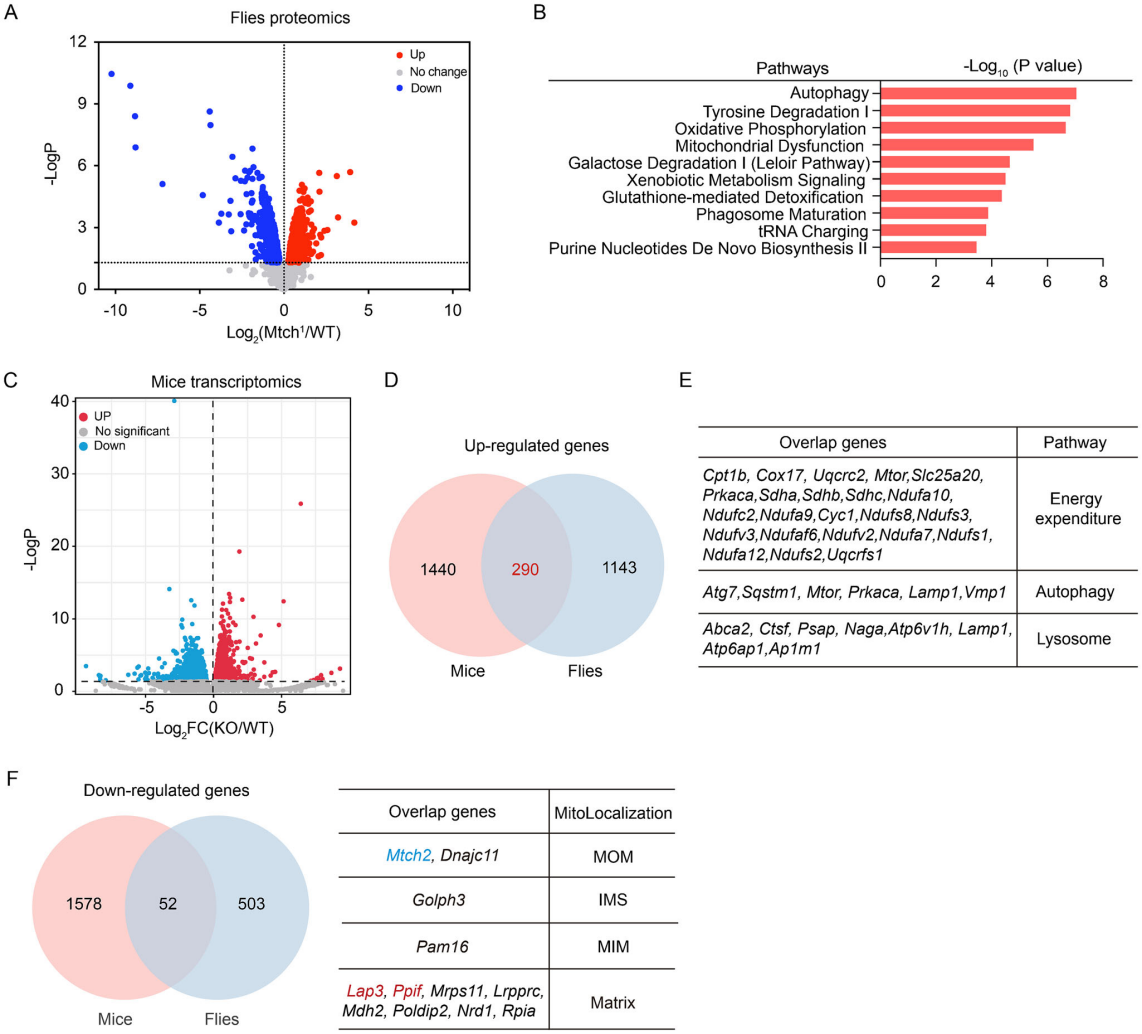
*Mtch2* deficiency promotes autophagy. (A) Volcano plot of differentially expressed proteins in *Mtch* mutant flies (fold change > 1 and *p* < 0.05). (B) Ingenuity pathway analysis (IPA) of up‐regulated proteins in *Mtch* mutant flies. (C) Volcano plot of differentially expressed genes in BAT of HFD‐fed *Mtch2^AKO^* mice (fold change > 1 and *p* < 0.05). (D) Venn diagram of the up‐regulated proteins in *Mtch* mutant flies and the up‐regulated genes in BAT of *Mtch2^AKO^* mice. (E) Functional enrichment analysis of the overlapped up‐regulated genes in flies and mice. (F) Venn diagram of the down‐regulated genes in BAT of *Mtch2^AKO^* mice and proteins in *Mtch* mutant flies (Left). The overlapped proteins localizing to the mitochondria were displayed (Right).

To gain a comprehensive and unbiased understanding of the underlying molecular mechanisms for the thermogenic role of *Mtch2* in adipose tissues, we employed a multi‐omics approach across flies and rodents, namely label‐free quantitative mass spectrometry in *Mtch*‐mutant flies, and RNA sequencing (RNA‐seq) on BAT from HFD‐fed *Mtch2^AKO^* mice. Mass spectrometry identified 1036 differentially expressed proteins between hypomorphic *Mtch* mutant and wildtype flies (**Figure** **5**A). Ingenuity Pathway Analysis (IPA) of differentially expressed proteins showed enrichment of autophagy, oxidative phosphorylation, and mitochondrial dysfunction pathways (Figure 5B). RNA‐seq analysis of BAT from *Mtch2^AKO^* and control mice identified 2491 differentially expressed genes (Figure 5C; Figure S5A, Supporting Information). KEGG pathway enrichment analysis identified upregulated genes involved in metabolic pathways, thermogenesis, lysosome, and autophagy in *Mtch2^AKO^* mice (Figure S5B, Supporting Information). A comparison of fly proteomics and BAT transcriptomic analyses in Venn diagrams indicated an overlap of 290 upregulated genes (Figure 5D). These genes were enriched in pathways related to energy metabolism, including energy expenditure, autophagy, and lysosome function (Figure 5E). This suggests that Mtch2 deficiency leads to a coordinated enhancement of cellular energy expenditure and metabolic efficiency. Specifically, the upregulation of autophagy‐related genes, such as *Atg7* in both fly and mouse datasets (Figure 5E) and *Atg5* and numerous other autophagy‐related genes in BAT transcriptome (Figure S5C, Supporting Information) reveals a potential link between thermogenesis, autophagy, and lysosome function. Among the downregulated genes with Mtch2 deficiency in the fly proteome and mouse BAT transcriptome, 52 were common in both datasets, with 12 identified in the MitoCarta Inventory^[^ ^19^ ^]^ (Figure 5F). Notably, leucine aminopeptidase III (*Lap3*) and peptidylprolyl isomerase F (*Ppif*, encodes Cyclophilin D, CypD), previously characterized as mitochondrial matrix proteins whose localization is facilitated by MTCH2, functioning as a insertase^[^ ^20^ ^]^‐have been implicated in fat metabolism and obesity,^[^ ^21^, ^22^, ^23^ ^]^ further supporting the MTCH2's critical role in regulating energy homeostasis.

### 2.6 Adipose Tissue‐Specific *Mtch2* Knockout Enhances Autophagy to Promote Lipolysis in Adipose Tissues

**Figure 6:**
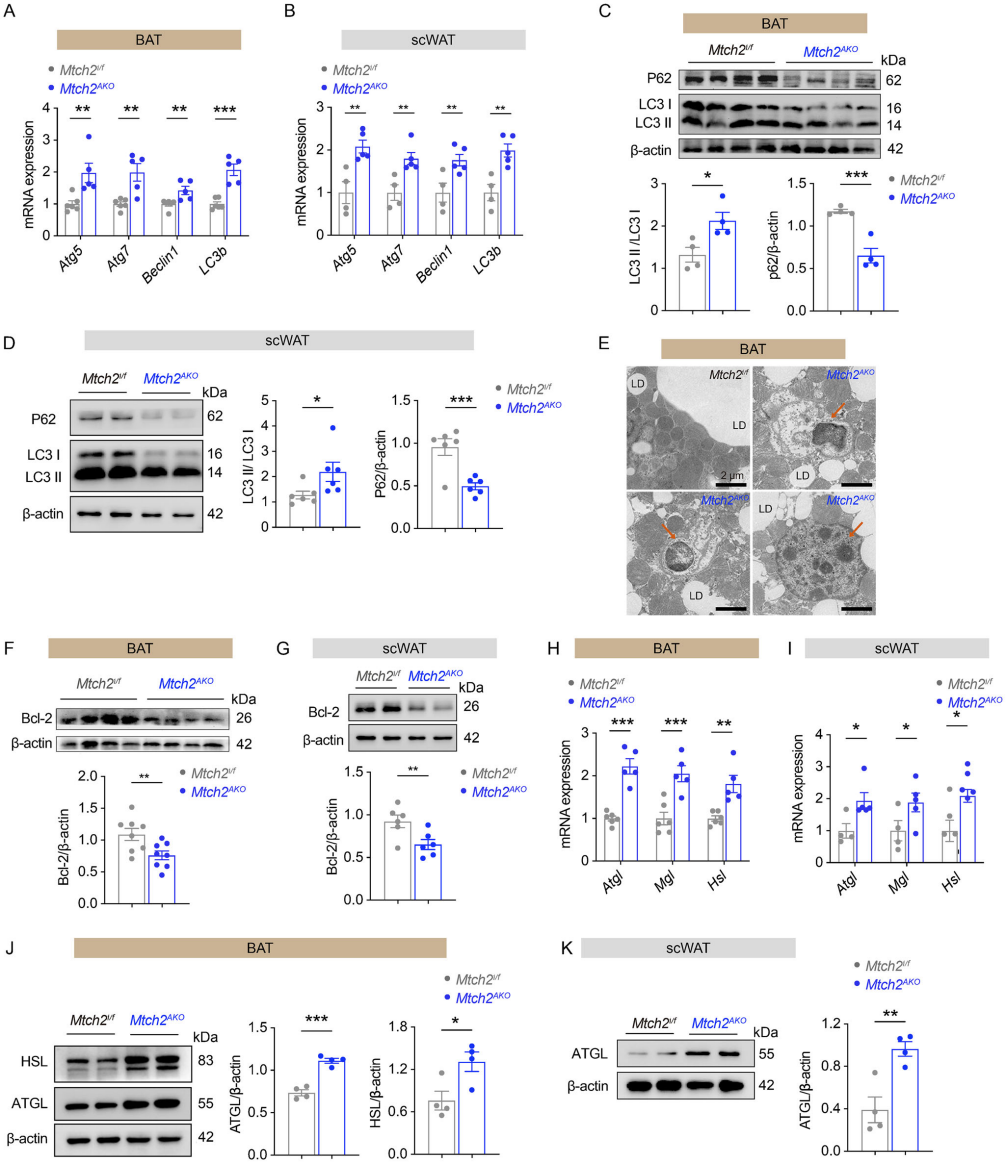
*Mtch2^AKO^* promotes autophagy and lipolysis in the adipose tissues in HFD‐fed mice. (A–D) Autophagy was induced in BAT (A,C) and scWAT (B,D) of *Mtch2^AKO^* mice by qPCR and western blot analysis of classic marker (*n* = 4–6). (E) The number of autophagosomes and autophagolysosomes was increased in BAT of *Mtch2^AKO^* mice by TEM analysis. (F,G) *Mtch2* knockout reduced Bcl‐2 protein levels in BAT (F) and scWAT (G) in 19 weeks of HFD‐fed mice (*n* = 6–8). (H–K) Lipolysis was increased in BAT (H,J) and scWAT (I,K) of *Mtch2^AKO^* mice by qPCR and western blot analysis of lipolytic gene and protein expression (*n* = 4–5). Data are represented as mean ± SEM. Two‐tailed unpaired *t*‐test was used. *p < 0.05, **p < 0.01 and ***p < 0.001, ns, not significant.

To validate the upregulation of autophagy pathways identified in both fly proteomic and mouse BAT RNA‐seq data, we conducted qPCR, western blot, and TEM analyses on BAT and scWAT. As expected, HFD‐fed *Mtch2^AKO^* mice exhibited significantly increased mRNA expression of key autophagy genes, including *Atg5*, *Atg7*, *Beclin1*, and *Lc3b*, compared to controls (**Figure** **6**A,B). Activation of autophagy facilitates the conversion of LC3I to LC3II and a decline in levels of the autophagy substrate p62. Here, we found elevated protein levels of LC3II/LC3I and reduced P62 in both BAT (Figure 6C) and scWAT (Figure 6D) of *Mtch2^AKO^* mice compared to control. TEM further revealed that the lack of *Mtch2* led to a notable increase in the number of autophagosomes and autophagolysosomes within BAT (Figure 6E), indicating enhanced autophagic activity. These results suggest that the autophagy pathway is enhanced in adipose tissue with Mtch2 knockout.

To explore the molecular mechanism underlying this enhanced autophagy, we focused on the potential regulators of autophagy. Bcl‐2 is a key suppressor of autophagy,^[^ ^24^, ^25^ ^]^ and Mtch2 is reported to interact with the Bcl‐2 family of proteins.^[^ ^26^, ^27^ ^]^ We investigated the possible involvement of Bcl‐2 in the suppression of autophagy by Mtch2 in adipose tissue. We observed a significant decrease in Bcl‐2 protein levels in both BAT (Figure 6F) and scWAT (Figure 6G) of *Mtch2* ^AKO^ mice compared with the control. Since autophagy has been implicated in promoting lipolysis in adipocytes^[^ ^28^, ^29^, ^30^ ^]^ and lipolysis promotes thermogenesis,^[^ ^3^, ^5^, ^31^ ^]^ we next examined changes in the expression of key lipolytic enzymes in adipose tissue following the loss of *Mtch2*. mRNA levels *Atgl*, *Hsl*, and *Mgl*, were significantly elevated in both BAT (Figure 6H) and scWAT of *Mtch2^AKO^* mice relative to controls (Figure 6I). Furthermore, we found elevated protein levels of HSL and ATGL in BAT (Figure 6J) and ATGL in scWAT (Figure 6K) of *Mtch2^AKO^* mice compared to control mice. These findings suggest that the deletion of Mtch2 increases lipolysis in adipose tissue. Collectively, these findings support the notion that Mtch2 deletion disinhibits autophagy, leading to increased lipolysis in adipose tissue, which in turn mediates the enhanced thermogenesis and energy expenditure under HFD conditions.

### 2.7 *Mtch2* Suppresses Thermogenesis Through the Bcl‐2‐Autophagy Pathway

**Figure 7:**
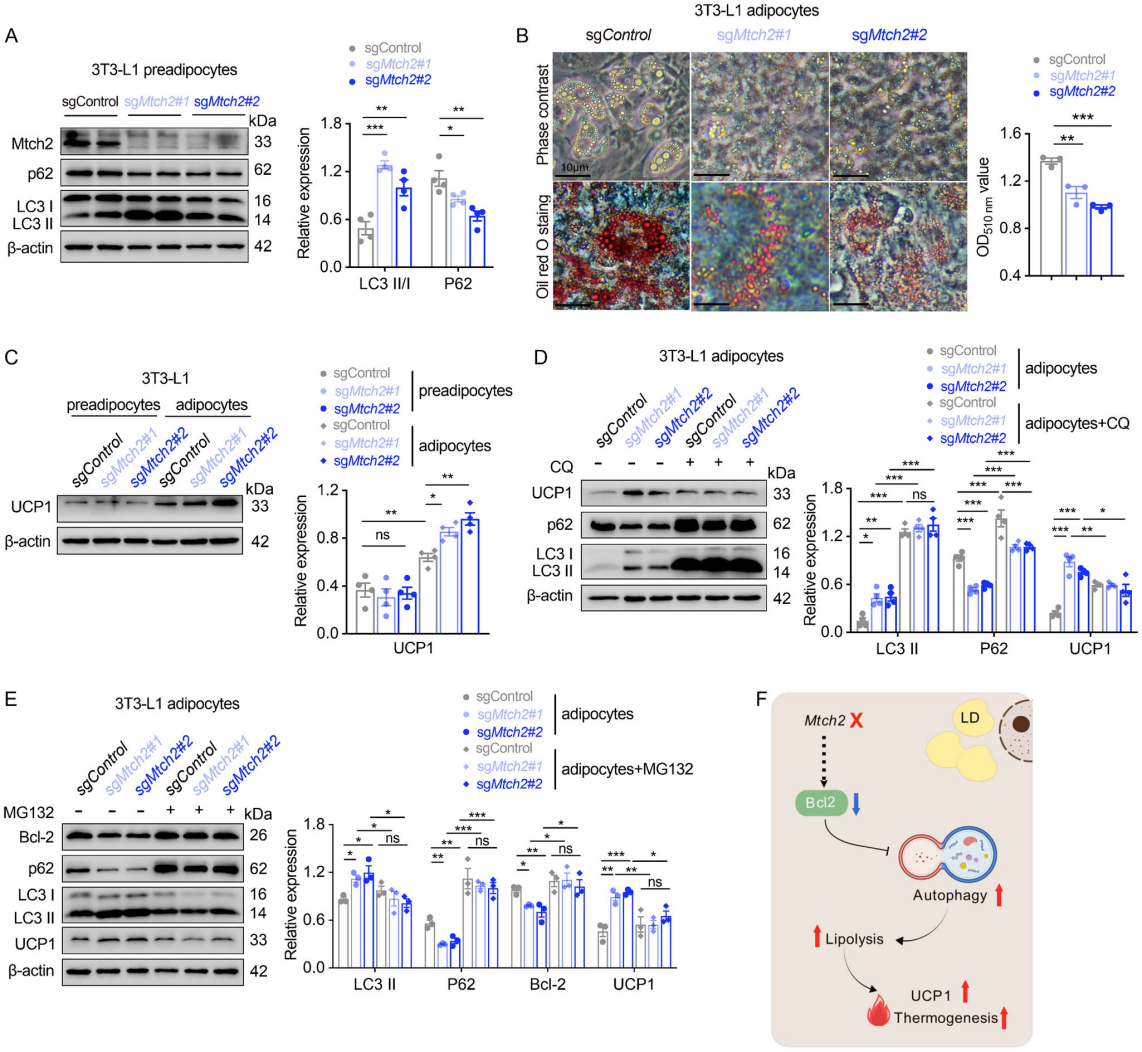
*Mtch2^AKO^* promotes thermogenesis through the Bcl‐2‐autophagy pathways. (A) Autophagy was increased in 3T3‐L1 preadipocytes following *Mtch2* knockout by CRISPR (*n* = 4). (B) TAG levels were reduced in 3T3‐L1 mature adipocytes following *Mtch2* knockout by CRISPR‐Cas9 technique (*n* = 3). (C) UCP1 protein levels were elevated in 3T3‐L1 preadipocytes and 3T3‐L1 mature adipocytes following *Mtch2* knockout by CRISPR (*n* = 4). (D) CQ treatment (50 µm) increased autophagy but reduced UCP1 protein levels in 3T3‐L1 mature adipocytes following *Mtch2* knockout by CRISPR (*n* = 4). (E) MG132 treatment (10 µm) increased Bcl‐2 protein levels but reduced UCP1 protein levels in 3T3‐L1 mature adipocytes following *Mtch2* knockout by CRISPR (*n* = 3). (F) A summary of MTCH2 suppresses thermogenesis via Bcl2‐mediated autophagy in adipose tissues. MTCH2 functions as a negative regulator of autophagy. MTCH2 knockout induces Bcl‐2 protein degradation to enhance autophagy, leading to increased lipolysis, elevated UCP1 protein levels, and promotion of BAT thermogenesis and scWAT browning (Created with BioGDP.com). Data are represented as mean ± SEM. Two‐tailed unpaired *t*‐test was used. *p < 0.05, **p < 0.01 and ***p < 0.001, ns, not significant.

To gain direct functional insights into the influence of Mtch2 on thermogenesis and autophagy in adipocytes, we utilized CRISPR/Cas9 technology to specifically delete *Mtch2* in mouse 3T3‐L1 preadipocytes, a widely used model system for analysis of adipocyte biology. Consistent with in vivo results in adipose tissues, *Mtch2* ablation led to changes in markers (increased LC3II/LC3I and reduced p62) indicative of increased autophagy in 3T3‐L1 preadipocytes (**Figure** **7**A). *Mtch2* deletion in mature 3T3‐L1 adipocytes resulted in a reduced lipid accumulation as revealed by Oil Red O staining and significantly lower levels of the eluted Oil Red O dye (Figure 7B). To investigate *Mtch2's* role in UCP1‐dependent thermogenesis, we measured UCP1 protein levels in both preadipocytes and mature adipocytes following *Mtch2* ablation. While UCP1 levels remained unchanged in preadipocytes, they were significantly increased in mature adipocytes (Figure 7C). These results with in vitro 3T3‐L1 adipocytes closely resemble the in vivo findings, suggesting that the enhancement of thermogenesis in mature adipocytes following *Mtch2* loss occurs in concert with increased UCP1.

We next examined whether changes in autophagic flux contribute to the heightened adipose thermogenesis following *Mtch2* inhibition. We treated control and *Mtch2*‐deleted mature 3T3‐L1 adipocytes with chloroquine (CQ), which inhibits autophagy flux by impairing autophagosome‐lysosome fusion.^[^ ^32^ ^]^ CQ treatment blocked the induction of UCP1 protein levels in *Mtch2*‐deleted adipocytes (Figure 7D), suggesting that *Mtch2* deletion promotes adipocyte browning and thermogenesis through activation of autophagy.

Reduced Bcl‐2 protein levels were associated with increased autophagy in MTCH2‐deficient adipose cells (Figure 6F,G). However, *Bcl‐2* mRNA expression remained unchanged in BAT and scWAT of *Mtch2^AKO^* mice compared to control mice (Figure S6A, Supporting Information), suggesting that *Mtch2* regulates Bcl‐2 protein levels via post‐transcriptional mechanisms, potentially involving pathways of protein stabilization or degradation processes. Co‐immunoprecipitation (Co‐IP) assay further revealed no direct interaction between Bcl‐2 and MTCH2 (Figure S6B, Supporting Information). MTCH2 is known to function as a membrane protein insertase that impacts cellular proteostasis,^[^ ^33^, ^34^, ^35^ ^]^ we examined whether the lack of MTCH2 enhances autophagy by promoting Bcl‐2 protein degradation in mature 3T1‐L1 adipocytes. To test this, we used the proteasome inhibitor MG132^[^ ^36^ ^]^ to block Bcl‐2 protein degradation. Remarkably, MG132 treatment restored Bcl‐2 protein levels and completely blocked the induction of autophagy in MTCH2‐deficient adipocytes, as evidenced by reduced LC3II and increased p62 levels (Figure 7E). In concert, MG132 treatment blocked the induction of UCP1 protein levels in MTCH2‐deficient adipocytes (Figure 7E).

Collectively, these findings reveal a previously unrecognized regulatory mechanism whereby MTCH2 represses thermogenesis in obesity. MTCH2 knockout in adipocytes results in Bcl‐2 protein degradation and disinhibition of autophagy, which leads to upregulation of UCP1 and increased thermogenesis (Figure 7F).

## 3 Discussion

Our research identifies a novel role for MTCH2 as a crucial regulator of adipose tissue biology through its control of mitochondrial adaptation and thermogenesis during obesity. Adipose‐specific knockout of MTCH2 protected against diet‐induced obesity and metabolic disorders, as evidenced by reduced fat deposition in adipose tissues and the liver, along with improved insulin sensitivity. These beneficial effects are mainly driven by enhanced energy expenditure, increased lipolysis, elevated BAT thermogenesis, and extensive being of scWAT linked to upregulation of UCP1. Mechanistically, adipose Mtch2 deletion increases mitochondrial biogenesis, and oxidative phosphorylation, primarily through heightened autophagic activity via the Bcl‐2 pathway. Our findings uncover MTCH2 as a key suppressor of adipose thermogenesis and establish a previously unrecognized link between the Mtch2‐Bcl‐2‐autophagy pathways in the regulation of thermogenesis and energy homeostasis. Thus, our studies suggest that the upregulation of adipose MTCH2 contributes to increased adiposity, disruption of thermogenesis, and metabolic disorder in obesity.

Mtch2 is enriched in WAT and its expression is elevated in WAT in obesity,^[^ ^37^ ^]^ suggesting a role in promoting adiposity and regulating lipid homeostasis. We found that mice lacking Mtch2 specifically in adipose tissues gained less body weight and fat mass with noticeably smaller adipocytes when fed an HFD. These results provide the first direct in vivo evidence that Mtch2 expression in adipose tissue is required for fat accumulation during dietary lipid oversupply. This was extended in vitro, where Mtch2 deletion by CRISPR/Cas9 in mature 3T3‐L1 adipocytes led to reduced lipid accumulation, consistent with previous findings.^[^ ^38^, ^39^ ^]^ Interestingly, prior studies suggest an obesogenic role of Mtch2 in other peripheral metabolically active tissues, with Mtch2 deletion in muscle^[^ ^16^ ^]^ and liver^[^ ^38^ ^]^ leading to reduced fat accumulation under HFD conditions. Thus, it is possible that targeting Mtch2 in multiple peripheral tissues may synergistically reduce lipid accumulation under HFD conditions.

Obesity arises from a chronic imbalance between food intake and energy expenditure. Our research found that adipose‐specific Mtch2 deletion did not affect food intake under either chow diet or HFD conditions. This finding is consistent with results from mice with liver‐specific Mtch2 modification.^[^ ^38^ ^]^ However, in contrast, increased food intake was observed in mice with Mtch2 deletion in either the forebrain (chow diet),^[^ ^40^ ^]^ or muscle (chow diet and HFD).^[^ ^16^ ^]^ Given that appetite and food intake are primarily controlled by the brain, particularly the hypothalamus,^[^ ^41^, ^42^ ^]^ it is likely that central Mtch2 plays a distinct role in controlling feeding behavior compared to peripheral Mtch2. The reason for increased food consumption in muscle‐specific Mtch2 deletion is unclear, but it may be a secondary effect stemming from muscle alterations, which could signal the hypothalamus to influence feeding. The complex regulation of feeding by Mtch2 warrants further investigation, particularly within the hypothalamus circuits that control feeding. Additionally, human studies have linked *MTCH2* gene variants to emotional eating,^[^ ^43^ ^]^ indicating MTCH2‐ particularly in the central nervous system – may play a role in integrating emotional states with feeding behavior.

The current study reveals that MTCH2 acts as a suppressor of energy expenditure in adipose tissue. A previous study also shows that MTCH2 control energy demand and expenditure to fuel anabolism during adipogenesis.^[^ ^44^ ^]^ The deletion of adipose Mtch2 increases whole‐body energy expenditure, largely through increased BAT thermogenesis and scWAT browning, driven by UCP1 upregulation. Although the mechanisms may differ, muscle‐specific Mtch2 knockout^[^ ^16^ ^]^ also increases energy expenditure in association with augmented mitochondrial biogenesis and oxidative phosphorylation in muscle. In our study, the enhanced BAT thermogenesis and scWAT beige observed with adipose deletion of Mtch2 was linked to greater heat production, increased whole‐body energy expenditure, and improved hepatic steatosis and insulin resistance under energy‐excess conditions. One limitation in our study is not assessing BAT activation by PET‐CT as previously described,^[^ ^45^ ^]^ though we employed multiple alternative methods instead. Moreover, the extensive upregulation of genes in BAT enriched in pathways related to energy metabolism, such as thermogenesis, autophagy, and lysosome function, indicates a coordinated response to enhance cellular energy expenditure and metabolic efficiency. The previously reported roles of Mtch2, as an outer mitochondrial membrane protein gatekeeper^[^ ^34^ ^]^ involved in mitochondrial dynamics and metabolism, including fusion, elongation, and regulation of gene transcription,^[^ ^35^, ^46^, ^47^ ^]^ highlight its importance in mitochondrial function and energy homeostasis. Additionally, Mtch2 has been implicated in promoting adipogenesis^[^ ^39^ ^]^ and inhibiting fat oxidation,^[^ ^38^ ^]^ further emphasizing its multifaceted roles in regulating lipid homeostasis. Together, our findings provide new insights into the complex regulation of lipid homeostasis by Mtch2, highlighting its potential as a target for metabolic intervention.

Our study reports an unidentified link between Mtch2, autophagy, and thermogenesis. Autophagy has been shown to be critical in lipid metabolism^[^ ^48^ ^]^ and thermogenesis.^[^ ^49^, ^50^, ^51^ ^]^ Additionally, autophagy regulates the initiation of lipolysis or lipophagy,^[^ ^52^ ^]^ and its activation promotes lipolysis and fatty acid oxidation.^[^ ^50^, ^53^ ^]^ In this study, *Mtch2* knockout activated autophagy, leading to increased lipolysis, upregulated UCP1 levels, and enhanced thermogenesis. Mouse RNA‐seq and fly proteomics analyses revealed marked upregulation of autophagy and lysosome pathways following Mtch2 deletion, which may promote cellular turnover and improve metabolic function by clearing damaged organelles and proteins. Overall, the changes point to an adaptation favoring increased energy utilization and cellular renewal, leading to mitigation of metabolic dysfunction in the context of overnutrition.

Mechanistically, Bcl‐2 is a well‐known repressor of autophagy^[^ ^24^, ^54^ ^]^ and a regulator of mitochondrial function.^[^ ^26^, ^27^, ^55^ ^]^ Elevated Bcl‐2 expression has also been linked to increased adiposity and body weight in mice.^[^ ^56^ ^]^ In this study, *Mtch2* knockout mitigated obesity and reduced Bcl‐2 protein levels, suggesting that Bcl‐2 may mediate metabolic benefit of *Mtch2* ablation via autophagy. Previous studies demonstrate that Bcl‐2 downregulation induces autophagy without compromising the mitochondrial functions,^[^ ^57^ ^]^ whereas Bcl‐2 upregulation^[^ ^58^ ^]^ or impaired degradation^[^ ^59^, ^60^ ^]^ suppresses autophagy. Our in vivo and in vitro findings reveal that while Mtch2 protein does not directly interact with Bcl‐2 protein, it post‐transcriptionally regulate Bcl‐2 protein stability, likely through stabilization or degradation mechanisms. These results broaden the understanding of how Mtch2 impacts cellular proteostasis,^[^ ^34^, ^35^ ^]^ particularly in maintaining Bcl‐2 protein levels to suppress autophagy, lipolysis, and energy expenditure in obesity. This highlights the critical role of Mtch2 in adipose tissue metabolism and energy homeostasis through mitochondrial and autophagic pathways. Notably, phosphorylation of Bcl‐2 at residues T69, S70, and S87 inhibits its anti‐autophagic function;^[^ ^24^, ^61^ ^]^ however, whether Mtch2 impacts Bcl‐2 phosphorylation remains unexplored. Further studies are needed to elucidate the precise mechanism by which Mtch2 controls Bcl‐2‐mediated autophagy thereby influencing thermogenesis and overall energy expenditure.

Obesity is linked to cognitive decline,^[^ ^62^ ^]^ and *MTCH2* variants have been associated with conditions such as Alzheimer's disease^[^ ^63^ ^]^ and emotional eating.^[^ ^43^ ^]^ That MTCH2 may be a critical player in neural cell biology was suggested in a study demonstrating mitochondrial dysfunction and impaired cognitive function in mice deficient in forebrain Mtch2.^[^ ^40^ ^]^ More work is necessary to elucidate the role of MTCH2 in both obesity and its related neurodegeneration. In addition, previous studies have shown a gender difference in MTCH2 expression, with protein levels significantly upregulated in women but not men with obesity.^[^ ^37^ ^]^ Future work is required to explore this gender difference, particularly in female mouse models. Importantly from a therapeutic perspective, pharmacological or gene‐based interventions aimed at modulating MTCH2 activity could offer promising strategies for treating metabolic disorders such as obesity and insulin resistance. However, due to its extensive distribution and complexity, careful evaluation of the systemic effects of MTCH2 manipulation is needed to avoid unintended consequences in tissues where MTCH2 may play a protective role.

In conclusion, our study identifies adipose MTCH2 as a key regulator of energy expenditure, with broad implications for understanding obesity and developing new treatments. By elucidating its role in modulating energy expenditure, lipid metabolism, and autophagy, we provide a solid foundation for the development of novel therapeutic strategies aimed at targeting MTCH2. Future research will continue to advance our understanding of Mtch2's mechanisms and potential to improve metabolic health.

## 4 Experimental Section

### Flies

All *Drosophila melanogaster* stocks were cultivated on a conventional medium comprising agar, sugar, and yeast, and were reared in an incubator at a constant temperature of 25 °C under a 12‐h light/dark period. The *Actin‐Gal4* driver line was sourced from the Bloomington *Drosophila* Stock Center. The RNAi fly lines utilized in this study were obtained from the *Vienna Drosophila Resource Center* (VDRC) and listed in Table S1 (Supporting Information). The flies were nurtured on a standard medium that included 1% agar, 3.6% yeast, 2% yellow corn meal, 5.4% sugar, and 3% molasses, and were also subjected to a 25 °C incubation environment with a 12‐h light/dark cycle. Following eclosion, adult male flies, aged 4–7 days post‐hatch, were selected for experimentation under various dietary conditions. For the experimental groups, flies were subjected to distinct diets. The normal diet consisted of 1% agar, 3.6% yeast, and 5.4% sucrose. The high sucrose diet was formulated with 1% agar, 3.6% yeast, and an elevated sucrose concentration of 16.2%. In contrast, the high‐fat diet contained 15% coconut oil, complemented by 1% agar, 3.6% yeast, and 5.4% sucrose.

### Mice

Heterozygous *Mtch2^flox/+^* mice were generated using CRISPR/Cas9 technology and obtained from Cyagen Biological Technology Co., Ltd. (Suzhou, China) on a C57BL/6J background. Homozygous *Mtch2^flox/flox^* mice were subsequently produced by intercrossing heterozygous pairs. To generate adipose tissue‐specific *Mtch2* knockout mice, *Mtch2^flox/flox^* mice were bred with mice expressing Cre recombinase under the control of the *Adiponectin* promoter. Genotyping was performed by PCR, with primer sequences listed in Table S2 (Supporting Information). Littermate mice of the same sex and age were utilized as controls for all experiments. All mice were maintained in an environment with ambient room temperature (23 ± 2 °C) and a 12‐h light/dark cycle, with unrestricted access to water and food, unless otherwise indicated. The experimental procedures involving mice were conducted by an Institutional Animal Care and Use Committee (IACUC)‐approved protocol from Sun Yat‐sen University, with the specific approval number SYSU‐IACUC‐2022‐000298.

### Cell and Cell Culture

Human embryonic kidney cell line HEK293T and the 3T3‐L1 preadipocyte cell line were both procured from the American Type Culture Collection (ATCC). The cells were maintained in Dulbecco's Modified Eagle Medium (DMEM, Life Technologies) supplemented with 10% (v/v) Fetal bovine serum (FBS) and 1% penicillin‐streptomycin. Cultivation was conducted at 37 °C in an atmosphere containing 5% CO_2_.

### Functional Analyses of *Drosophila*

Initiating crosses by mating ten virgin *Actin‐Gal4* females with 5–7 young males of either UAS‐RNAi or control w1118 strains to induce RNAi‐mediated whole‐body knockdown of the target genes. Upon eclosion of the F1 progeny, male flies were isolated and, at 4–7 days of age, assigned to either a normal diet or a high sucrose diet for 10 days. Body weight was ascertained using an analytical balance. To assess triglyceride levels, a group of ten male flies was weighed, homogenized in 200 µL of ice‐cold PBST (phosphate‐buffered saline with 0.05% Tween 20), and sonicated. Subsequently, 800 µL of ice‐cold PBST was added to the homogenate and mixed thoroughly. A 50 µL aliquot of this mixture was utilized to quantify triglycerides with the Roche triglycerides assay kit, following the manufacturer's guidelines. Triglyceride measurements were normalized relative to body weight. The data were standardized to a Z‐score employing the formula: 𝑍 = (𝑋−𝜇)/𝜎, where 𝑋 is the individual's value, 𝜇 is the control group mean, and σ is the standard deviation of the control group. The threshold for statistical significance was set at a 95% confidence interval for a two‐tailed test, equating to a Z‐score threshold of ≥ 1.96 or ≤ −1.96.^[^ ^64^ ^]^ Target genes identified as hits were validated through three independent screening assays for confirmation.

### Protein Mass Spectrometry

Protein mass spectrometry analysis was conducted following the methods outlined in a previous report.^[^ ^65^ ^]^ In summary, the proboscis or the entire body of the specimen was extracted and homogenized in a lysis buffer containing 6 m urea, 2 m thiourea, 25 mm TEAB, and 0.1% SDS. The homogenate was then subjected to sonication, followed by centrifugation at 15 000 g for 10 min at ambient temperature. The resulting supernatant was subjected to precipitation overnight with pre‐chilled acetone. The precipitated protein pellets were subsequently resuspended in a buffer comprising 6 m urea, 2 m thiourea, and 25 mm TEAB. Protein reduction was performed using 10 mm DTT at 30 °C for 60 min, followed by alkylation with 25 mm IAA for 30 min at room temperature in darkness. The protein mixture was then enzymatically digested with LysC (enzyme‐to‐substrate ratio of 1:100) at 30 °C for 2 h. Post‐digestion, the protein solution was diluted 1:5 with 25 mm TEAB and further digested with trypsin (enzyme‐to‐substrate ratio of 1:50) at 37 °C for an extended period. The resultant peptide mixture underwent desalting via stage‐tips and was subsequently lyophilized using vacuum centrifugation in preparation for total proteome analysis.

### Mass Spectrometry Analysis and Data Processing

MS‐based total proteome analysis was performed on an Easy nLC‐1000 UHPLC coupled to a Q‐Exactive in positive polarity mode as in the previous report.^[^ ^65^ ^]^ The in‐house packed C18AQ column (75 µm × 40 cm, 1.9 µm particles) separated the peptides using a 360‐min gradient of 10–35% acetonitrile (ACN) containing 0.1% formic acid (FA) at a flow rate of 200 nL min^−1^ and a column temperature of 55 °C. The MS1 scan encompassed a range of 300–1750 m/z with a resolution of 70 000, AGC target of 3e6, and injection time of 100 ms. Data‐dependent MS/MS acquisition subsequently targeted the top 20 ions using HCD fragmentation with a resolution of 17 500, AGC target of 5e5, injection time of 60 ms, normalized collision energy (NCE) of 25, and an isolation window of 2.0 m/z. Raw data processing employed MaxQuant (v1.5.3.25) against a UniProt Drosophila database. Minor adjustments were made to the default settings: oxidation of methionine and N‐terminal protein acetylation were designated as variable modifications, while cysteine carbamidomethylation was set as a fixed modification. Re‐quantification, second peptide searches, and match‐between‐runs features were enabled. Peptide spectral match (PSM) and protein false discovery rate (FDR) were both set to 1%. The “Label‐free Quantification (LFQ) method” was employed for protein quantification and normalization. Bioinformatics analysis, primarily conducted using the LIMMA package within the R programming environment, aimed to identify differentially expressed proteins. Functional annotation was performed using DAVID v6.8 beta (https://david‐d.ncifcrf.gov/).

### Gene Analysis in Clinical Cohorts

The initial transcriptomics data were derived from abdominal subcutaneous white adipose tissue (WAT) biopsies of 25 male and 28 female individuals, generated as previously reported. These transcriptome profiles were acquired using the Affymetrix Human Gene 1.1 ST Array, and the data have been deposited in the NCBI Gene Expression Omnibus (GEO) under the accession code GSE77962.^[^ ^66^ ^]^ Subsequent transcriptome profiles were sourced from subcutaneous WAT biopsies of 770 male participants in the METSIM study, utilizing the Affymetrix Human Genome U219 Array, with the dataset archived under accession code GSE70353.^[^ ^67^ ^]^ Additional transcriptomics data were procured from abdominal subcutaneous adipose needle biopsies of 56 women, employing the Affymetrix Human Gene 1.0 ST Array, and registered with the accession code GSE25401.^[^ ^68^ ^]^ In these analyses, obesity was defined by a BMI exceeding 30 kg m^−^^2^.

### Body Weight

Male mice were fed either a normal chow diet (NCD) from four weeks of age or a high‐fat diet (HFD; Research Diets, D12492, 60% kcal from fat, 20% kcal from carbohydrate, 20% kcal from protein, 5.42 kcal g^−1^) starting at nine weeks of age for the indicated experimental periods. Body weight was monitored weekly at a consistent time point throughout the study.

### Intraperitoneal Glucose Tolerance Test (IPGTT) and Insulin Tolerance Test (ITT)

IPGTT and ITT were performed at the indicated time points. Mice were fasted for 15 h (18:00‐09:00) before IPGTT or for 6 h (09:00‐15:00) before ITT. Glucose (2.0 g kg^−1^ body weight) or insulin (0.75 U kg^−1^ body weight) was administered intraperitoneally, and blood glucose levels were measured using a digital glucometer (Yuwell, Jiangsu Yuyue Medical Equipment & Supply Co., LTD, China) at baseline and 15, 30‐, 60‐, 90‐, and 120‐min post‐injection.

### Food Intake

Spontaneous food intake was measured after a 24‐h acclimation period in individual cages with ad libitum food access, as previously described.^[^ ^1^ ^]^ Equal food quantities were provided to each mouse at the start of the measurement period. Food remnants and spillage were collected and weighed after 24 h. Food intake was calculated by subtracting the combined weight of remnants and spillage from the initial food amount.

### Core Temperature and Surface Temperature

Core body temperature was measured rectally in conscious mice at specified time points following cold exposure (4 °C) using an electronic thermometer (FT3400, DEJR, China). Surface temperature was assessed using thermal imaging with a Fluke Ti480U infrared thermal camera (Fluke Corporation, USA) after 2 h of cold exposure (4 °C).

### Energy Metabolism by Indirect Calorimetry

Mice were acclimatized to Promethion Metabolic Systems (Sable Systems International, North Las Vegas, NV, USA) for 24 h before data collection. Oxygen consumption, carbon dioxide production, respiratory exchange ratio, energy expenditure, and locomotor activity were continuously monitored for 48 h. Oxygen consumption, carbon dioxide production, and energy expenditure were normalized to body weight as previously described.^[^ ^1^, ^69^ ^]^

### Body Composition

Body composition, including fat and lean mass, was assessed in mice using nuclear magnetic resonance (NMR) spectroscopy (Small Animal Body Composition Analysis and Imaging System, MesoQMR23‐060H‐I, Suzhou Niumag Corporation, Shanghai, China) according to the manufacturer's instructions.

### Tissue Collection

Mice were euthanized by intraperitoneal injection of pentobarbital sodium (50 mg kg^−1^). Following euthanasia, adipose tissues (BAT, scWAT, eWAT, and rWAT) and liver were carefully excised, weighed, snap‐frozen in liquid nitrogen, and stored at −80 °C for subsequent analysis.

### Hepatic Triglyceride Measurements

≈20 mg of snap‐frozen tissue was homogenized in 180 µL of ice‐cold absolute ethanol and subsequently centrifuged at 2500 rpm for 10 min at 4 °C. A 2.5 µL aliquot of the supernatant was used to quantify hepatic triglycerides using the GOP/PAP method according to the manufacturer's instructions.

### Plasma Parameters Measurement

Plasma ALT and AST were determined using a Beckman CX5 automated biochemical analyzer (Beckman Coulter, Inc., United States) according to standard operating procedures of the Guangdong Engineering & Technology Research Center for Disease‐Model Animals, Sun Yat‐sen University.

### RNA Extraction and qPCR

Total RNA was extracted from tissues or cells using TRIzol reagent. Subsequently, 1 µg of RNA was reverse transcribed into cDNA using a PrimeScript RT reagent kit according to the manufacturer's protocol. Real‐time quantitative PCR (qPCR) was performed using SYBR Green dye on a QuantStudio 5 system (Applied Biosystems, Thermo Fisher Scientific). Gene expression levels were normalized to the housekeeping gene, 18S ribosomal RNA (Rn18S),^[^ ^70^ ^]^ using the ΔΔCt method and expressed as fold change relative to controls. Primer sequences are listed in Table S2 (Supporting Information).

### RNA‐Sequencing and Data Analysis

RNA sequencing was outsourced to BerryGenomics. Before library preparation, RNA quality from BAT was assessed using an Agilent TapeStation. Sequencing libraries were constructed using the TruSeq RNA Sample Preparation Kit v2 (Illumina) according to the manufacturer's instructions. Library quality and fragment size were validated using a 2100 Bioanalyzer (Agilent). Normalized libraries were then pooled for sequencing on an Illumina NovaSeq 6000 platform using barcoded multiplexing for single‐end reads of 150 bp. RNA sequencing data analysis was performed using OmicShare tools, a freely accessible online platform^[^ ^71^ ^]^ (https://www.omicshare.com/tools).

### Mtch2 mRNA Expression in Mice

Twelve‐week‐old male wide‐type C57BL/6J mice were euthanized, and the tissues were carefully excised to extract RNA. Adipose tissues of obese mice and same‐aged lean mice were obtained from the previous research.^[^ ^1^ ^]^ *Mtch2* mRNA expression was analyzed by qPCR.

### Mitochondrial DNA (mtDNA) Copy Number Analysis

Relative mtDNA copy number analysis was performed as previous report.^[^ ^72^ ^]^ Briefly, genomic DNA from adipose tissue was extracted using a TIANamp Genomic DNA Kit. Quantitative PCR was performed using QuantStudio 5 (Applied Biosystems, Thermo Fisher Scientific) to quantify mtDNA and nuclear 18S rRNA. The ΔΔCt method was used to calculate the relative mtDNA copy number, normalized to 18S rRNA. Primer sequences are listed in Table S3 (Supporting Information).

### Western Blot

Tissue lysates were prepared using RIPA buffer (Hangzhou FuDe Biological Technology Co., LTD, China) supplemented with protease and phosphatase inhibitors (including 1 mm PMSF). Total protein lysates were boiled with a loading buffer containing 10% SDS‐PAGE. Soluble proteins (30 ug) were separated on a 10% or 12.5% SDS polyacrylamide gel and transferred onto a 0.2 µm PVDF membrane (Immobilon‐PSQ, Merck). After blocking with 5% non‐fat milk in TBST buffer (containing 0.1% Tween 20 in 1xTBS) for 1 h at room temperature, membranes were incubated with primary antibodies overnight at 4 °C. Membranes were then rinsed three times for 10 min each time in TBST and were incubated with an HRP‐conjugated secondary antibody for 1 h at room temperature. Antibody α‐tubulin (Ray antibody Cat#RM2007), β‐actin (Servicebio Cat#GB15003), UCP1 (Abcam Cat#ab209483), HSL (ABclonal Cat#A15686), ATGL (ABclonal Cat#A5126), LC3A/B (Cell Signaling Technology Cat#12741), SQSTM1/p62 (Servicebio Cat#GB11531), Bcl‐2(ABclonal Cat#A19693), MTCH2(Absin Cat#abs143485), Goat anti‐rabbit IgG HRP (Ray Antibody Cat#RM3001) and Goat anti‐mouse IgG HRP (Ray Antibody Cat#RM3002) were used. Immunoblotted bands were detected using enhanced chemiluminescence (ECL) reagent, imaged by a BG‐gdsAUTO 720 Imaging system (Baygene, Beijing), and quantified by densitometry using Fiji software (NIH). The intensity of proteins of interest was normalized to the intensity of the loading control.

### Co‐Immunoprecipitation (Co‐IP) Assays

Co‐IP was performed using Immunoprecipitation Kit (Beyotime, Cat #P2179S) according to the manufacturer's protocol. 3T3L1 cells were washed with PBS for three times and lysed on ice for 30 min. One mg cellular proteins were then incubated with 1 µg primary antibodies at 4 °C overnight on the roller. 20 µL Protein A/G‐Magnetic Beads were later added to capture antigen‐antibody complex at 4 °C for 2 h. Subsequently, the samples were washed for three times with magnetic grate, suspended with loading buffer, and boiled at 95 °C for 5 min. The supernatant was collected for Western blot analysis with 20 µL supernatant per well.

### Histological Analysis and Immunohistochemistry

Adipose tissues and liver tissues were fixed in 10% neutral buffered formalin, embedded in paraffin, and sectioned at a 4 µm thickness. Hematoxylin and eosin (H&E) staining of the resulting sections was performed according to the manufacturer's instructions. Adipocyte size was analyzed by Fiji software (NIH) as previously reported.^[^ ^17^ ^]^ Immunohistochemical staining of the resulting sections for UCP1 was performed with a rabbit monoclonal antibody (Abcam Cat#ab209483) at 1:2000 dilution. Antigen retrieval was conducted using an EDTA solution. The images were captured using a Nikon microscope.

### Transmission Electron Microscope

BAT tissues from 19‐week HFD‐fed mice were dissected into 1 mm^3^ cubes and fixed in 2.5% glutaraldehyde prepared in sodium cacodylate buffer (2 h at room temperature, followed by 12 h at 4 °C). Subsequent processing involved post‐fixation in 1% osmium tetroxide, staining with 2% uranyl acetate, dehydration in an ethanol series (30–100%), and embedding in EMbed‐812 resin. Ultrathin sections (50–60 nm) were stained with uranyl acetate and lead citrate before imaging on a Hitachi H600 electron microscope (Hitachi, Japan) at 120 kV.

### Mtch2 Knockout in 3T3‐L1 Cells by CRISPR/Cas 9

3T3‐L1 cells were infected with a second‐generation lentiviral system packaged in 293T cells to generate *Mtch2* knockout (KO) cells as previous report.^[^ ^73^ ^]^ Lentiviral vectors expressing sgRNA targeting *Mtch2* (sgRNA 1#: TACCGGGAAGAAGGCATCGT; sgRNA 2#: CTTTCA CGTACATGAGCGGC) were constructed in pLentiCRISPRv2 and transduced into 3T3‐L1 cells. Puromycin selection was used to enrich KO cells, which were subsequently validated by Western blot.

### Adipocyte Differentiation

3T3‐L1 preadipocytes were induced to differentiate two days post‐confluence (day 0). The differentiation medium contained insulin (5 µg mL^−1^), dexamethasone (1 µm), 3‐isobutyl‐1‐methylxanthine (0.5 mm), indomethacin (125 nm), triiodothyronine (1 nm), and rosiglitazone (1 µm). From day 2, the medium was replaced with a maintenance medium containing insulin (5 µg mL^−1^), triiodothyronine (1 nm), and rosiglitazone (1 µm), which was changed every other day. Fully differentiated adipocytes were harvested on day 6.

### Oil Red O Staining

Oil Red O staining was performed as previously reported.^[^ ^74^ ^]^ Oil Red O (0.5% (mg/v) in isopropanol) was diluted with water (3:2), and then filtered through a 0.45 mm filter. The differentiated adipocytes were washed three times with PBS and fixed for 30 min with 4% (v/v) paraformaldehyde (PFA), then the fixed cells were incubated with filtered Oil Red O for 1 h at room temperature to visualize lipid droplets. After being washed with PBS, 100% isopropanol was added as an extraction solution to extract the staining dye of cells. The absorbance of the extracted dye was measured at 510 nm using a BioTek Epoch2 microplate reader.

### Statistical Analysis

Data are presented as Mean ± S.E.M. Statistical analyses were performed using GraphPad Prism 9. Unpaired *t*‐tests, one‐way ANOVA with Bonferroni post hoc test, or two‐way ANOVA with Bonferroni post hoc test were used as appropriate. Differences were considered significant at *p < 0.05, **p < 0.01, ***p < 0.005, or ****p < 0.001.

## Conflict of Interest

The authors declare no conflict of interest.

## Author Contributions

X.Y.Z., Q.P.W., and G.G.N. conceptualized the study. X.Y.Z. and Q.P.W. developed the methodology. X.Y.Z., B.C.Z., H.L.L., Y.L., B.W., and A.Q.L. conducted the investigation. X.Y.Z., B.C.Z., H.L.L., and Q.P.W. performed the validation. X.Y.Z., A.Q.L., J.X.M., and Q.P.W. created the visualization. X.Y.Z. and Q.P.W. wrote the original draft. X.Y.Z., T.S.Z., H.X.Y.H., J.S., D.C., N.G., J.H., J.X.M., D.R.L., Y.Y.D., Y.C.S., G.G.N., and Q.P.W. reviewed and edited the manuscript. Q.P.W. and Y.C.S. acquired funding. Q.P.W. and G.G.N. provided resources. G.G.N., Y.C.S., and Q.P.W. supervised the study.

## Supporting information



Supporting Information

## Data Availability

The data that support the findings of this study are available from the corresponding author upon reasonable request.
