# Supplementary material for: MTCH2 Suppresses Thermogenesis by Regulating Autophagy in Adipose Tissue
Source: Adv Sci (Weinh). 2025 Mar 7;12(17):2416598. doi: 10.1002/advs.202416598 (PMC12061245; doi:10.1002/advs.202416598)
Supplement: Supplementary file 1 — Supporting Information [file ADVS-12-2416598-s001.doc]

**Supplemental information**

**MTCH2 Suppresses Thermogenesis by Regulating Autophagy in Adipose Tissue**

*Xin-Yuan Zhao, Ben-Chi Zhao, Hui-Lin Li, Ying Liu, Bei Wang, An-Qi Li, Tian-Shu Zeng, Hannah Xiaoyan Hui, Jia Sun,* *Domagoj Cikes, Nele Gheldof,* *Jorg Hager,* *Jian-Xun Mi, D Ross Laybutt, Yin-Yue Deng,* *Yan-Chuan Shi*, G Gregory Neely*, Qiao-Ping Wang**

**
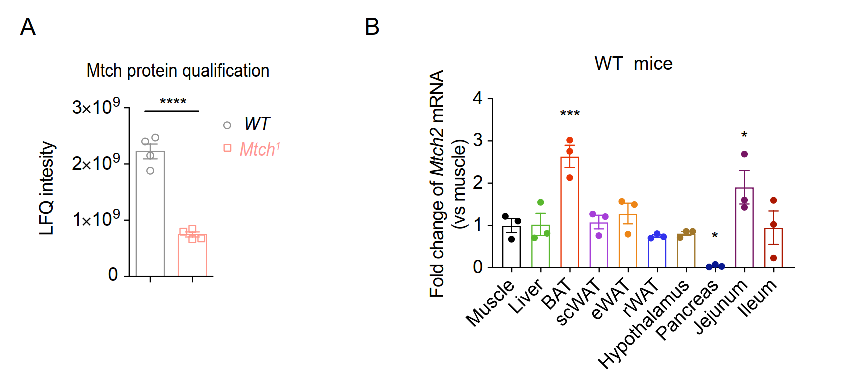
Figure S1 Identification of MTCH2 as a conserved regulator of energy homeostasis.**

(A) *Mtch* protein levels were reduced by 70% in *Mtch* mutant flies (n = 4 biological samples).

(B) *Mtch2* was expressed in different tissues in chow-fed C57BL/6J mice at the age of 12 weeks (n=3).

Data are represented as mean ± SEM. Two-tailed unpaired Student’s t-test (A), and One-way analysis of variance (ANOVA) with Tukey’s multiple comparisons test (B) were used. **p* < 0.05, ***p* < 0.01, ****p* < 0.005, *****p* < 0.001.

**
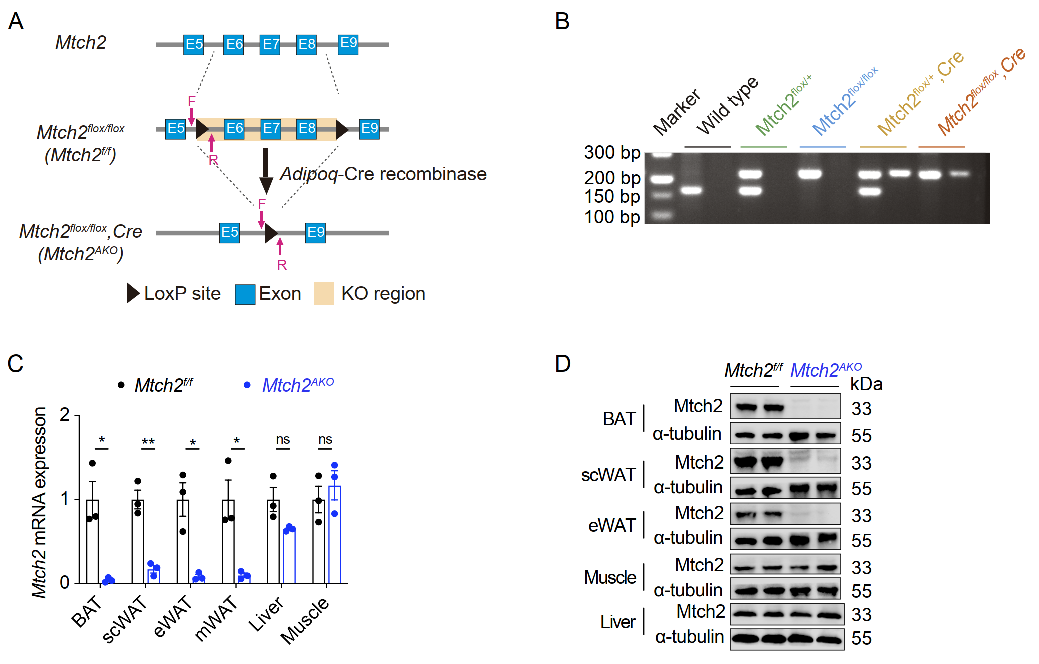
**

**Figure S2 Generation and validation of adipose tissues *Mtch2* specifically knockout mouse**

(A) A schematic diagram of generating Flox-Cre mediated *Mtch2* specifically knockout in adipose tissues in mice (*Mtch2^flox/lox^, Adipoq-Cre, Mtch2^AKO^*). The region of exon 6-8 in DNA was deleted.

(B) Adipose tissue-specific *Mtch2* knockout was confirmed by genomic PCR.

(C-D) Adipose tissue-specific *Mtch2* knockout was confirmed by qPCR of *Mtch2* mRNA (C, n=3) and western blot of Mtch2 protein (D, n=2) in the liver, muscle, and adipose tissues of *Mtch2^AKO^* mice.

Data are represented as mean ± SEM. Two-tailed unpaired Student’s t-test was used (C) **p* < 0.05, ***p* <0.01, and ****p* < 0.001, ns, not significant.


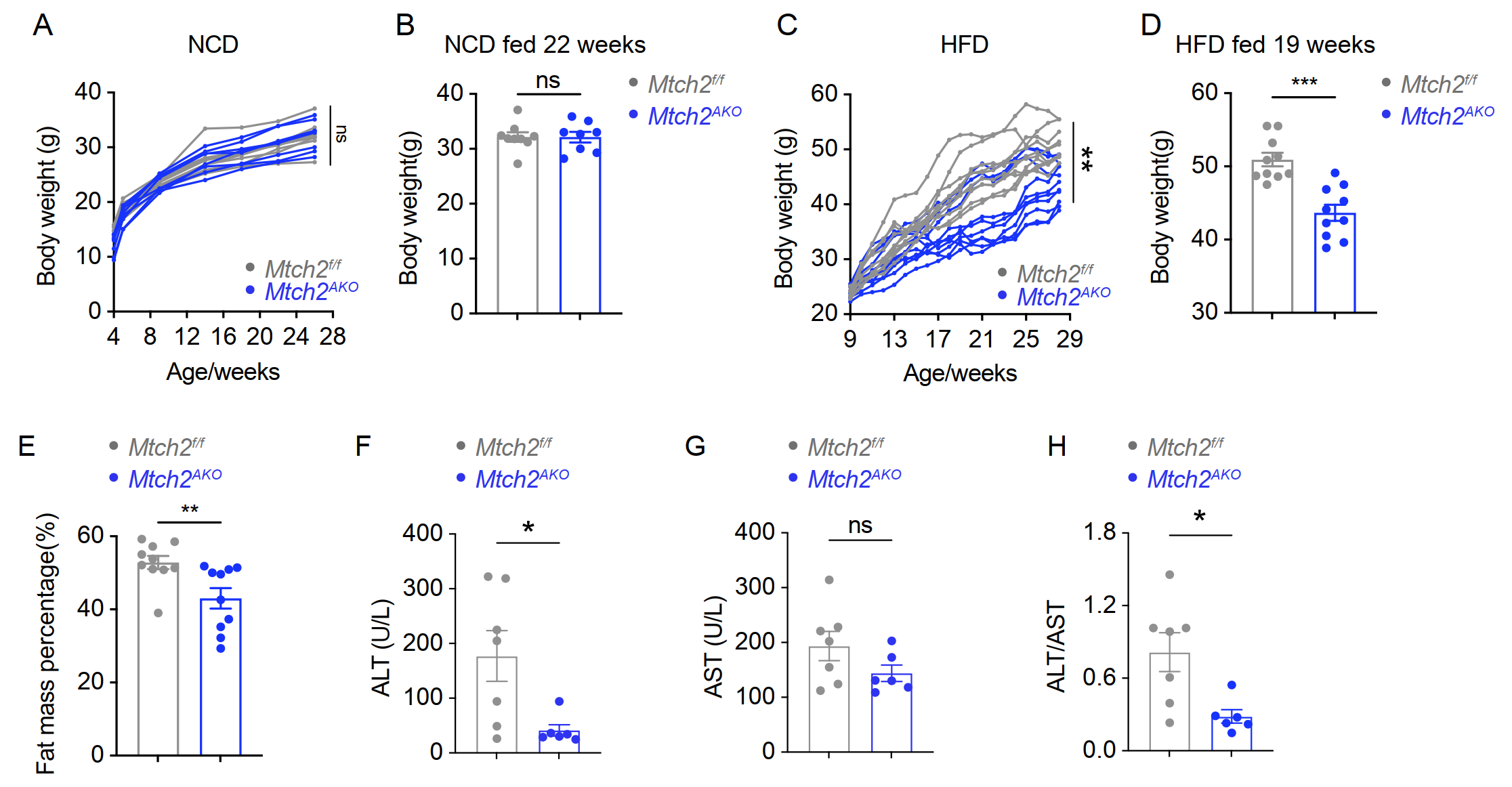


**Figure S3 *Mtch2^AKO^* reduces fat depots and hepatic accumulation under HFD.**

(A-B) *Mtch2^AKO^* did not affect body weight in mice fed chow. Body weight curve of individual mice (A) and body weight at 26 weeks of age under chow diet (B) (n = 8-9).

(C-D) Body weight was gained less in *Mtch2^AKO^* mice under HFD feeding for 19 weeks. Body weight curve of individual mice (C) and body weight at 28 weeks of age under HFD (D) (n = 10).

(D) *Mtch2^AKO^* mice displayed a lower fat composition. Fat mass percentage by NMR (D) (n = 10).

(E-H) *Mtch2^AKO^* protected mice from HFD induced liver injury. Plasma ALT and ALT/AST were reduced, plasma AST was not significant changed in *Mtch2^AKO^* mice (n = 6-7).

Data are represented as mean ± SEM. Two-way ANOVA followed by Bonferroni’s multiple comparisons test (A, C), Two-tailed unpaired t-test was used (B, D, E-H), **p* < 0.05, ***p* < 0.01, and ****p* < 0.001, ns, not significant.

**
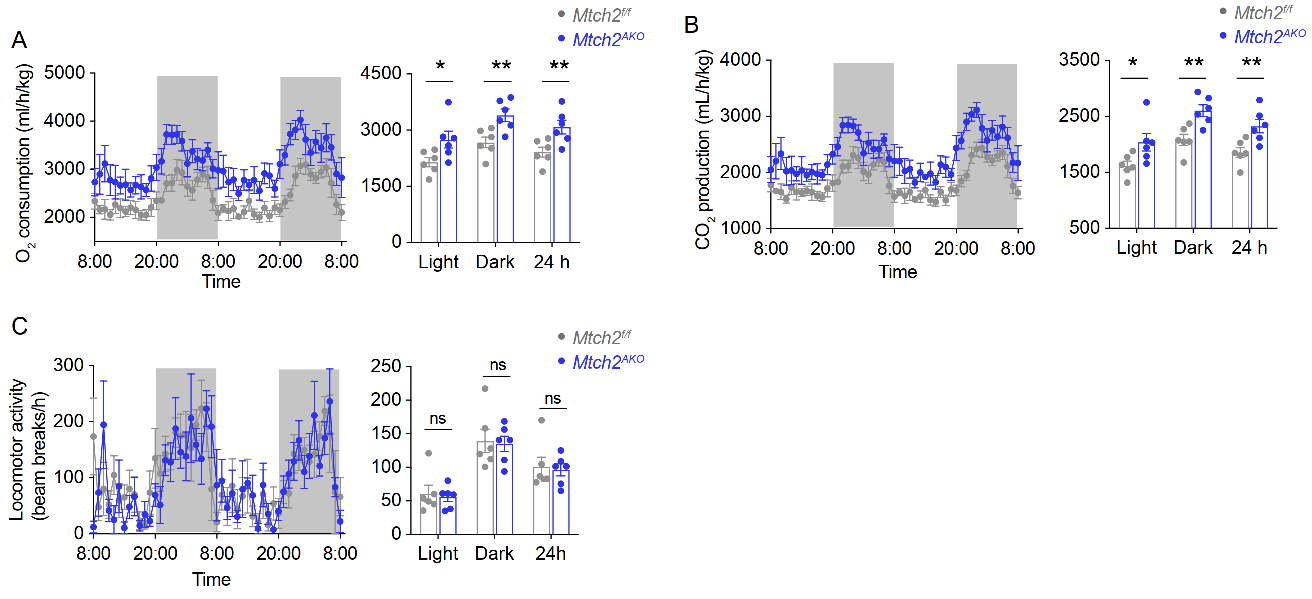
Figure S4 *Mtch2^AKO^* increases oxygen consumption and carbon dioxide production in HFD-fed mice.**

(A-B) Oxygen consumption (A) and carbon dioxide production (B) were increased in HFD-fed *Mtch2^AKO^* mice (n = 6).

(C) Locomotor activity was not affected in HFD-fed *Mtch2^AKO^* mice (n = 6).

Data are represented as mean ± SEM. Two-tailed unpaired Student’s t-test was used **p* < 0.05, ***p* <0.01, and ****p* < 0.001, ns, not significant.

**
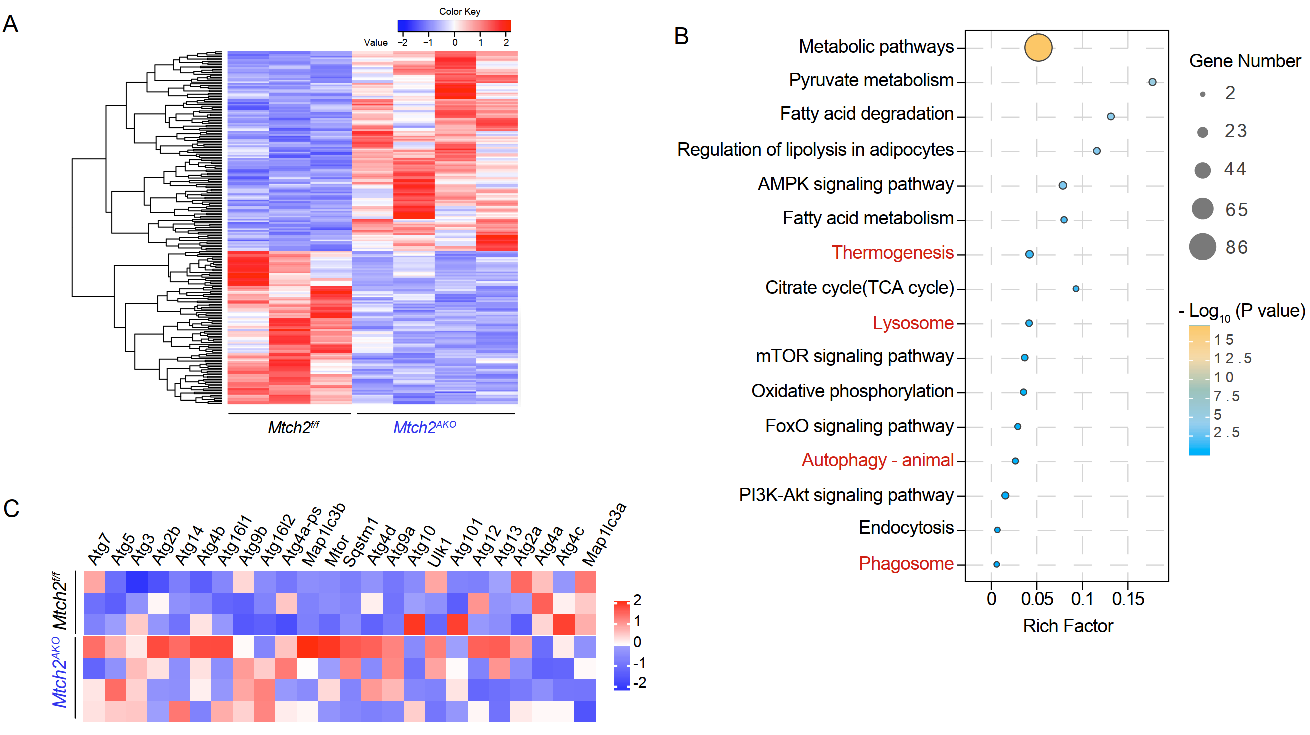
**

**Figure S5 *Mtch2^AKO^* increases thermogenesis and autophagy in BAT in HFD-fed mice**

(A) Heatmap of significantly differentially expressed genes in BAT of *Mtch2^AKO^* mice. Colors indicate the log2 values of normalized read counts.

(B) KEGG pathways enrichment of upregulated genes in BAT of *Mtch2^AKO^* mice.

(C) The expression of the autophagy-related gene was elevated in BAT of *Mtch2^AKO^* mice.

**
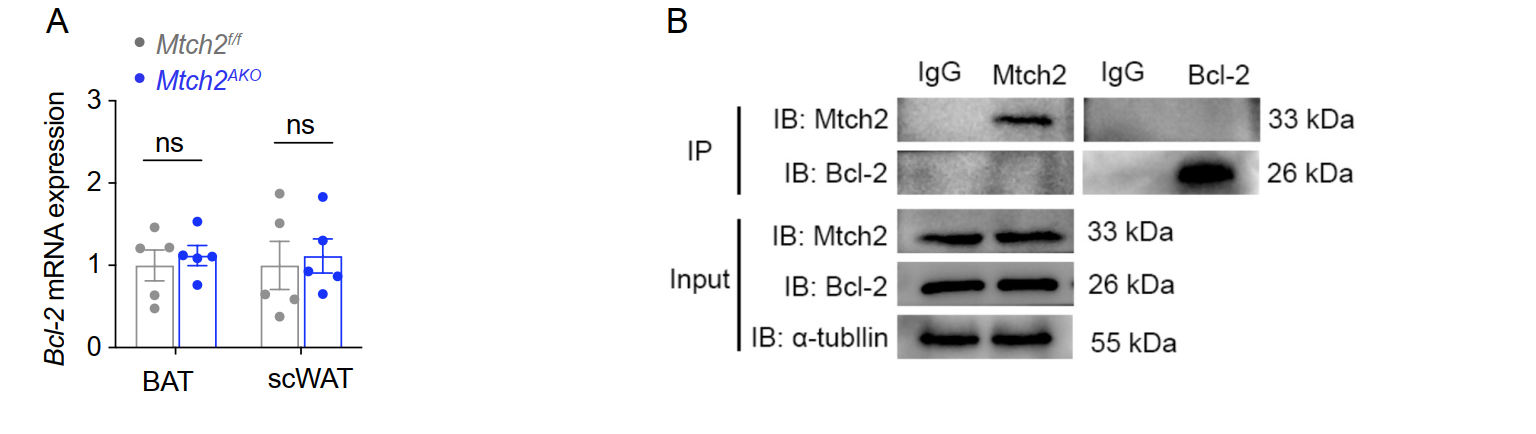
**

**Figure S6 *Mtch2* does not affect the mRNA expression of *Bcl-2* and interact with Bcl-2**

(A) Bcl-2 mRNA expression was not changed in BAT and scWAT of *Mtch2^AKO^* mice by qPCR analysis (n=5).

(B) There is no direct interaction between MTCH2 and Bcl-2 protein by Co-IP analysis.

Data are represented as mean ± SEM. Two-tailed unpaired Student’s t-test was used (A) **p* < 0.05, ***p* <0.01, and ****p* < 0.001, ns, not significant.

**Table legends**

Table S1. The information of *Drosophila* melanogaster

| *Drosophila* lines | Source | IDENTIFIER |
| --- | --- | --- |
| *D. melanogaster: w1118* | Qiaoping Wang lab. | N/A |
| *D. melanogaster*: *Act-Gal4/cyo* | Bloomington | BDSC:4414 |
| *D. melanogaster*: *Men* RNAi | VDRC | VDRC: 104016 |
| *D. melanogaster*: *Men-b* RNAi | VDRC | VDRC: 27535 |
| *D. melanogaster*: *Menl-1* RNAi | VDRC | VDRC: 50645 |
| *D. melanogaster*: *SPARC* RNAi | VDRC | VDRC: 16677 |
| *D. melanogaster*: *Mtch* RNAi | VDRC | VDRC: 44305 |
| *D. melanogaster*: *CG10920* RNAi | VDRC | VDRC: 16092 |
| *D. melanogaster*: *Loxl1* RNAi | VDRC | VDRC: 16619 |
| *D. melanogaster*: *Wun* RNAi | VDRC | VDRC: 6446 |
| *D. melanogaster*: *Wun2* RNAi | VDRC | VDRC: 4176 |
| *D. melanogaster*: *CG11426* RNAi | VDRC | VDRC: 42599 |
| *D. melanogaster*: *CG11438* RNAi | VDRC | VDRC: 108551 |
| *D. melanogaster*: *Laza* RNAi | VDRC | VDRC: 42594 |
| *D. melanogaster*: *CG11437* RNAi | VDRC | VDRC: 9452 |
| *D. melanogaster*: *CG11425* RNAi | VDRC | VDRC: 8458 |
| *D. melanogaster*: *Elo68β* RNAi | VDRC | VDRC: 30043 |
| *D. melanogaster*: *Elo68α* RNAi | VDRC | VDRC: 9206 |
| *D. melanogaster*: *Elovl7* RNAi | VDRC | VDRC:48139 |
| *D. melanogaster*: *CG31522* RNAi | VDRC | VDRC: 37329 |
| *D. melanogaster*: *Sit* RNAi | VDRC | VDRC: 43091 |
| *D. melanogaster*: *Ser* RNAi | VDRC | VDRC: 27172 |
| *D. melanogaster*: *CG31999* RNAi | VDRC | VDRC: 29155 |
| *D. melanogaster*: *Lam* RNAi | VDRC | VDRC: 45636 |
| *D. melanogaster*: *Lkr* RNAi | VDRC | VDRC: 22845 |
| *D. melanogaster*: *Octβ1R* RNAi | VDRC | VDRC: 47895 |
| *D. melanogaster*: *TyrR* RNAi | VDRC | VDRC: 2857 |
| *D. melanogaster*: *LpR1* RNAi | VDRC | VDRC: 106364 |
| *D. melanogaster*: *LpR2* RNAi | VDRC | VDRC: 25684 |
| *D. melanogaster*: *Hsp26* RNAi | VDRC | VDRC: 100955 |
| *D. melanogaster*: *Hsp23* RNAi | VDRC | VDRC: 102493 |
| *D. melanogaster*: *Hsp67Ba* RNAi | VDRC | VDRC: 21806 |
| *D. melanogaster*: *Hsp22* RNAi | VDRC | VDRC: 43632 |
| *D. melanogaster*: *l(2)efl* RNAi | VDRC | VDRC: 40532 |
| *D. melanogaster*: *CG7409* RNAi | VDRC | VDRC: 40637 |
| *D. melanogaster*: *Hsp27* RNAi | VDRC | VDRC: 40530 |

Table S2. Primer sequences for *Mtch2 LoxP* and *Adipoq-Cre* mice genotyping

| Primer name | Primer sequences (5’to 3’) |
| --- | --- |
| *LoxP* | Forward: GACTAAAGCTCTAAGGTGCCACAC  Reverse: GAGGCATCAGGTCTAGATGAGACA |
| *Adipoq-Cre* | Forward: GAACGCACTGATTTCGACCA  Reverse: GCTAACCAGCGTTTTCGTTC |

Table S3. Primer sequences of mice in Q-PCR

| Mouse genes | Primer sequences (5’to 3’) |
| --- | --- |
| *Rn18s* | Forward: GTAACCCGTTGAACCCCATT  Reverse: CCATCCAATCGGTAGTAGCG |
| *Mtch2* | Forward: GGGAAAGTCTTACAGTATTACCAGG  Reverse: ATGAGGGTAGCAGCAGAACG |
| *Ucp1* | Forward: AGGCTTCCAGTACCATTAGGT  Reverse: CTGAGTGAGGCAAAGCTGATTT |
| *Cidea* | Forward: TGACATTCATGGGATTGCAGAC  Reverse: GGCCAGTTGTGATGACTAAGAC |
| *Dio2* | Forward: AATTATGCCTCGGAGAAGACCG  Reverse: GGCAGTTGCCTAGTGAAAGGT |
| *Elovl3* | Forward: TCCGCGTTCTCATGTAGGTCT  Reverse: GGACCTGATGCAACCCTATGA |
| *Cox7a1* | Forward: GCTCTGGTCCGGTCTTTTAGC  Reverse: GTACTGGGAGGTCATTGTCGG |
| *Atgl* | Forward: GGATGGCGGCATTTCAGACA  Reverse: CAAAGGGTTGGGTTGGTTCAG |
| *Mgl* | Forward: ACCATGCTGTGATGCTCTCTG  Reverse: CAAACGCCTCGGGGATAACC |
| *Hsl* | Forward: CCAGCCTGAGGGCTTACTG  Reverse: CTCCATTGACTGTGACATCTCG |
| *Pgc1α* | Forward: TATGGAGTGACATAGAGTGTGCT  Reverse: CCACTTCAATCCACCCAGAAAG |
| *Tfam* | Forward: CTGTTCCGGGGAATGTGGAG  Reverse: CTGATAGACGAGGGGATGCG |
| *Nrf1* | Forward: AATGACCCAGGCTCAGCTTC  Reverse: GCTTGCAGCTTTCTTTCCCC |
| *Ndufs8* | Forward: GACTGGGCATGACCCTAAGTT  Reverse: CGCTCCTCTCCAGATGGGTA |
| *Sdhb* | Forward: AATTTGCCATTTACCGATGGGA  Reverse: AGCATCCAACACCATAGGTCC |
| *Uqcrc1* | Forward: ATCAAGGCACTGTCCAAGG  Reverse: TCATTTTCCTGCATCTCCCG |
| *Atp5a1* | Forward: TCATTTTCCTGCATCTCCCG  Reverse: TCCCAAACACGACAACTCC |
| *Atg5* | Forward: TAGAATATATCAGACCACGACG  Reverse: CTCCTCTTCTCTCCATCTTC |
| *Atg7* | Forward: TCCGTTGAAGTCCTCTGCTT  Reverse: CCACTGAGGTTCACCATCCT |
| *Beclin1* | Forward: GGCCAATAAGATGGGTCTGA  Reverse: GCTGCACACAGTCCAGAAAA |
| *LC3b* | Forward: ACAAAGAGTGGAAGATGTCCGGCT  Reverse: TGCAAGCGCCGTCTGATTATCTTG |
| *18S ribosomal DNA* | Forward: AAACGGCTACCACATCCAAG  Reverse: CAATTACAGGGCCTCGAAAG |
| *mtDNA* | Forward: CCCCAGCCATAACACAGTATCAAAC  Reverse: GCCCAAAGAATCAGAACAGATGC |
